# Research Progress on Microstructure Control and Strengthening Mechanisms of Aluminum Alloys for New Energy Vehicles

**DOI:** 10.3390/ma19132880

**Published:** 2026-07-06

**Authors:** Jia-Shuai Chu, Hong-Yu Yang, Bai-Xin Dong, Tian-Shu Liu, Shi-Li Shu, Jia Meng, Cheng-Gang Wang, Pei-Jun Cong, Hong-Jun Zhang, Jian Qiao, Feng Qiu, Qi-Chuan Jiang

**Affiliations:** 1 National Key Laboratory of Automotive Classis Integration and Bionics, Jilin University, Changchun 130025, China; chujs24@mails.jlu.edu.cn (J.-S.C.); tsliu97@suda.edu.cn (T.-S.L.); jiangqc@jlu.edu.cn (Q.-C.J.); 2 Key Laboratory of Automobile Materials, Ministry of Education and Department of Materials Science and Engineering, Jilin University, Renmin Street No. 5988, Changchun 130025, China; 3 School of Mechanical and Aerospace Engineering, Jilin University, Renmin Street No. 5988, Changchun 130025, China; 4 Department and Test Center, FAW-Volkswagen Automotive Co., Ltd., Changchun 130011, China; jia.meng@faw-vw.com; 5 Technology Research and Development Casting & Forging Research Institute, FAW Foundry Co., Ltd., Crossing of Hexie Street & Bingwu Road Automotive Industry Development Zone, Changchun 130013, China; cgwang0611@163.com (C.-G.W.); 15555699450@163.com (P.-J.C.); 6 Dazheng Metal (Changchun) Co., Ltd., Factory Building in Axle Gear C Zone, FAW Foundry Park, Foundry Road, Automobile Development Zone, Changchun 130013, China; 19556732870@163.com; 7 School of Mechatronic Engineering and Automation, Foshan University, No. 33 Guangyun Road, Nanhai District, Foshan 528231, China; qiaojj99065@163.com

**Keywords:** Al-Si-Mg alloys, Al-Mg-Si alloys, integrated die-casting, nanoparticle strengthening, microstructural control, strengthening mechanisms

## Abstract

Against the backdrop of rapid advancements in lightweighting and integrated die-casting technologies for new energy vehicles, aluminum alloys have emerged as the core lightweight material for automotive structural components due to their excellent specific strength, corrosion resistance, and formability. However, traditional die-cast aluminum alloys generally rely on heat treatment processes to achieve strength and toughness. This not only significantly increases process costs and production lead times, but also leads to critical defects such as blistering and dimensional distortion in large integrated die-castings during heat treatment. Consequently, the development of high-strength and high-toughness die-cast aluminum alloys that meet service requirements in their as-cast state has become an urgent industry need. Although significant progress has been made in recent years in the research on microstructural control and performance enhancement of die-cast aluminum alloys for automotive applications, a systematic, integrated analysis of the strengthening mechanisms for the two mainstream alloy systems—Al-Si-Mg and Al-Mg-Si—remains lacking. This paper systematically reviews the microstructural evolution patterns and intrinsic mechanisms of performance enhancement for these two alloy systems, providing important theoretical references for the composition design and microstructural control of next-generation high-strength, high-toughness aluminum alloys without subsequent heat treatment, as well as supporting the further development of lightweighting technologies for new energy vehicles.

## 1. Introduction

**Figure 1:**
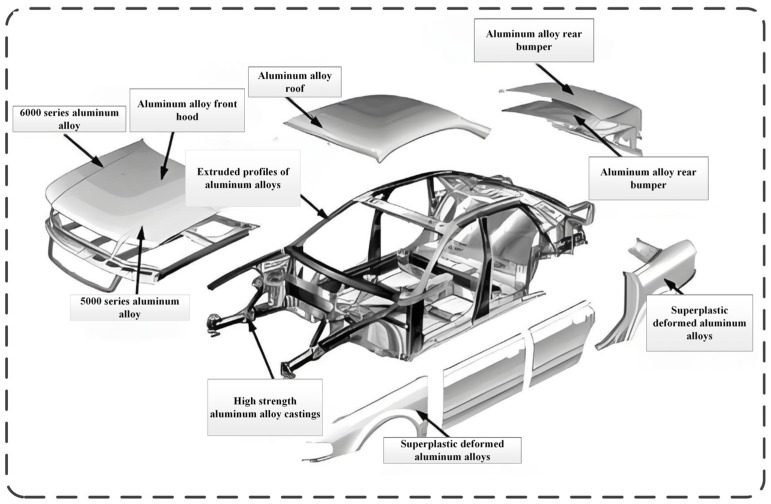
Aluminum alloys designed for structural utilization in new energy vehicles.

The automotive industry is undergoing a profound transformation. While advances in internal combustion engines can expand vehicle production, the associated emissions have concurrently exacerbated environmental concerns. Consequently, new energy vehicles have proliferated rapidly, with major manufacturers launching dedicated models. Unlike conventional fuel-powered vehicles, new energy vehicles eliminate the internal combustion powertrain and fuel storage systems. However, to address range limitations, current technology primarily relies on large-capacity battery packs, which keep overall vehicle weight comparable to that of conventional fuel-powered vehicles. Therefore, the lightweight production of structural components has become a central focus in automotive design. Aluminum alloys have gradually become a key material in the automotive industry due to their excellent specific strength and favorable casting properties [1,2,3,4,5,6,7,8,9]. Figure 1 illustrates the use of aluminum alloys in automotive components. Traditional cast aluminum alloys heavily rely on heat treatment to achieve their mechanical properties, as it promotes the formation of strengthening precipitates that enhance strength and toughness. However, such heat treatment is energy-intensive and costly, and it often induces deformation and residual stresses. Particularly in large and thin-walled castings, it severely compromises dimensional accuracy [10].

The automotive industry has generated unprecedented demand for aluminum alloy manufacturing technologies, driving a comprehensive technological revolution fueled by the need to extend the driving range of new energy vehicles. To reduce vehicle weight, automakers are seeking to use components with higher levels of integration. For certain large, structurally complex monolithic die-cast components, it is nearly impossible to enhance strength through traditional heat treatment processes [11]. Their massive thermal mass and complex geometries inevitably lead to unacceptable deformation and extremely high energy consumption; furthermore, the choice of cooling medium during heat treatment also affects their performance [12]. Therefore, it has become essential to develop a material that requires no heat treatment yet meets mechanical property requirements in the as-cast state. These alloys rely primarily on revolutionary alloy composition design and precisely controlled casting processes. By introducing specific trace elements, they directly form fine, uniform strengthening phases and microstructures during solidification. This enables them to achieve comprehensive as-cast properties that match or even surpass those of conventional heat-treated alloys. Alloying, as the most fundamental and effective method for strengthening aluminum alloys, fundamentally manipulates the microstructure of aluminum alloys through the careful design of alloying elements and their proportions, thereby significantly enhancing their strength, toughness, and service performance. The essence of alloying lies in the introduction of alloying elements whose differences in size, modulus, and charge drive a series of microstructural evolutions. The primary strengthening mechanisms include solution strengthening, precipitation strengthening, and secondary strengthening. For example, Cu, Mg, Zn, and Si phases can dissolve into an aluminum matrix to induce lattice distortion and form nanoscale coherent/semi-coherent precipitation phases through heat treatment, thereby producing precipitation strengthening. This constitutes the primary source of strength in aluminum alloys such as Al-Cu, Al-Si-Mg, Al-Mg-Si, and Al-Zn-Mg [10,13]. Xue et al. [14] examined the precipitation behavior and mechanical properties of Al8Si0.5Mg and Al8Si2Cu0.5Mg alloys. They observed the formation of β” phase during peak aging. The motivation for developing particle-reinforced alloys stems from their ability to deliver a more comprehensive set of properties than conventional metals [15,16,17]. For example, Liu et al. [18] discovered that incorporating TiC particles can slow solidification rates, reduce segregation, suppress grain coarsening, and promote more uniform distribution of secondary phases, thereby enhancing the mechanical properties of alloys. Furthermore, particle reinforcement can synergize with microalloying to yield superior material performance. However, an excessive particle content may cause agglomeration, which not only fails to improve material properties but can also degrade alloy performance.

Consequently, elucidating the mechanisms by which various alloying elements and particles modify microstructures and enhance properties is of critical importance. This research provides valuable insights for developing more advanced die-cast aluminum alloys. Beginning with the emergence of integrated die-casting and the consequent need for die-cast aluminum alloys, this review systematically examines how alloying elements and particle reinforcements tailor the microstructure and performance of Al-Si-Mg/Al-Mg-Si alloys. This review concludes by providing a reference for the design of next-generation die-cast aluminum alloys.

## 2. Research Status of Die-Cast Aluminum Alloys

In response to stringent greenhouse gas regulations and fuel efficiency requirements, the automotive industry is increasingly leveraging lightweight aluminum alloys, exploiting their favorable mechanical properties and low density [19]. HPDC serves as the key manufacturing technology, widely employed across automotive and aerospace for its excellent performance. Traditional cast aluminum alloys typically require post-casting heat treatment to improve performance [20]. Among them, T6 treatment is not suitable for large thin-walled castings because severe distortion often occurs during solution treatment, leading to part deformation. During the rapid injection stage of HPDC, gas can become trapped in the molten metal. The residual gas expands at high temperatures and forms bubbles, which prevents most of the castings, especially large thin-walled castings from being heat-treated [21]. As a result, traditional aluminum alloys are unable to meet the needs of integral die-casting, creating an urgent need for alloys that satisfy their mechanical properties without heat treatment. Currently, die-cast aluminum alloys have been widely studied and used for high-pressure die-casting of thin-walled components [22]. These alloys are characterized by high strength and toughness in the as-cast state, offering significant production advantages, with elongation often exceeding 10%. The following Table 1 and Table 2 list common die-cast aluminum alloy grades in the Al-Si and Al-Mg series and their alloy compositions.

**Figure 2:**
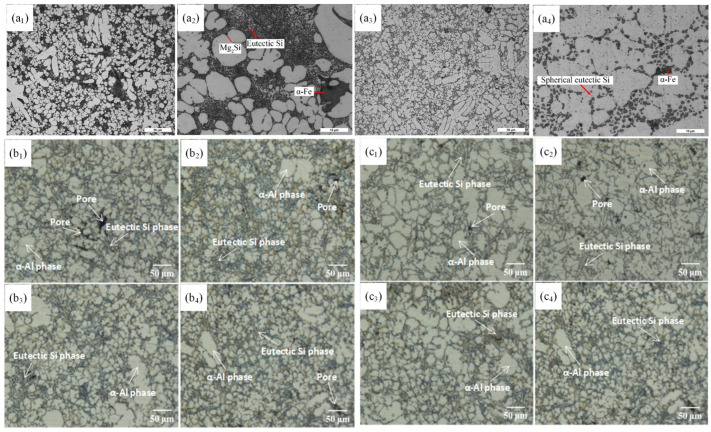
The microstructure of AlSiMgMn alloy: (**a_1_**,**a_2_**) as-cast; (**a_3_**,**a_4_**) T6 heat treatment [30]. Microstructure of thick-walled area at different injection speeds: (**b_1_**) 3.5 m/s, (**b_2_**) 4.0 m/s, (**b_3_**) 4.5 m/s, (**b_4_**) and 5.0 m/s. Microstructure of thick-walled area at different boost pressures: (**c_1_**) 700 bar, (**c_2_**) 750 bar, (**c_3_**) 800 bar, and (**c_4_**) 850 bar [31].

**Figure 3:**
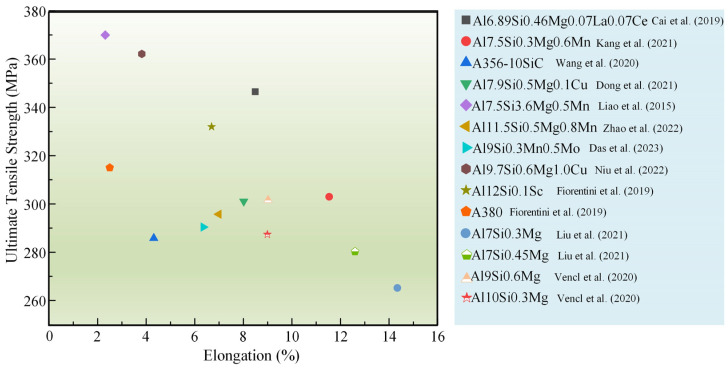
A summary of the mechanical properties of selected die-cast aluminum alloys [8,36,37,38,39,40,41,42,43,44,45].

Driven by the rapid advancement of IDC, die-cast aluminum alloys have emerged as the primary material for producing complex, lightweight components. Die-cast aluminum alloys are particularly important for integrated die-casting technology and have attracted extensive research attention. Key aluminum alloy systems for die-casting are favored for their balanced castability and mechanical properties. Al-Si series die-casting aluminum alloys have good fluidity, a narrow crystallization temperature range, high latent heat and specific heat during silicon-phase solidification, and low thermal shrinkage, making them the most widely applied. Al-Mg series die-casting alloys maintain high strength while also exhibiting good toughness and superior resistance to stress corrosion. Al-Si-Cu series alloys have high strength and hardness along with excellent casting processability. Al-Si-Mg aluminum alloys show good fluidity and are well-suited for complex-shaped parts. Through heat treatment, especially artificial aging (T5 or T6 treatment), significant strength improvements can be achieved. One of the major challenges remaining is finding materials with excellent ductility that are required for manufacturing ultra-large thin-walled castings by HPDC. Liu et al. [30] investigated the microstructure of AlSiMgMn alloys under high-vacuum conditions. The figure shows α-Al grains, Mg_2_Si phases, and eutectic phases, with the eutectic Si particles appearing in fibrous or short rod-like forms. After T6 treatment, the eutectic Si particles were significantly fragmented (Figure 2(a_1_–a_4_)). Die-casting process parameters also have a significant impact on the microstructure of die-cast aluminum alloys. Peng et al. [31] investigated the effects of casting pressure and casting speed on the microstructure of A380 aluminum alloy castings. As shown in the figure, as the casting speed increases, the average grain size, eutectic Si phase, and shrinkage cavities gradually decrease (Figure 2(b_1_–b_4_)). The figure also shows that as the casting pressure increases, the average grain size, eutectic Si phase, and shrinkage cavities also gradually decrease (Figure 2(c_1_–c_4_)). By incorporating rare earth elements (La and Ce), Peng et al. [32] developed an enhanced Al-Si-Mg die-casting aluminum alloy. The microstructure of the die-cast aluminum alloy exhibits a fine coral-like structure. Dong et al. [10] developed Al-Si-Mg aluminum alloys through big-data-driven machine learning. The microstructures of three typical die-casting alloys are depicted in Figure 3 in the paper by Dong et al. The constituent phases in Al-Si-Mg die-casting alloys include α-Al, eutectic Si, Mg_2_Si, and α-Fe, the latter primarily located at grain boundaries and in eutectic regions. Figure 6 in the paper by Dong et al. presents tensile stress–strain curves for as-cast die-casting alloys with varying Si/Mg ratios. A clear trade-off can be observed: strength and plasticity are inversely correlated, with a higher alloying content generally increasing strength but diminishing ductility. In HPDC, when the superheated melt contacts the cooler cavity walls, extensive α-Al nucleates and grows near the cavity walls due to injection delay, forming externally solidified crystals (ESCs). Larger ESC dendrites may fracture during filling due to melt impact. A lower filling temperature leads to a mixture of ESCs, fine α-Al grains, and solute-enriched liquid [33,34,35]. Under applied pressure, this creates a specific microstructure. The corresponding mechanical properties are summarized in Figure 3 for the evaluated alloys.

In recent years, integrated die-casting technology (IDCT) has advanced rapidly, with die-cast aluminum alloys emerging as an ideal material for producing complex-shaped components [44,45]. Currently, most commercial aluminum–silicon alloys suffer from limited mechanical properties and poor machinability, and the majority of aluminum alloys are produced through heat treatment, which incurs additional costs. Aluminum alloys for die-casting represent a novel class of materials that require no solution or artificial aging treatment, making them a key component for integrated die-casting. The table below lists the designations and nominal compositions of commonly used heat-treatable Al-Si-based die-casting alloys. Al-Si alloys exhibit excellent casting, achieving complete filling, and are one class of several casting aluminum alloys widely used in industrial production. For instance, Castasil37 is an Al-Si series die-casting aluminum alloy developed by the Rhein-Feld Company in Germany. Its key feature is that the increased Mg content enhances strength, achieving a YS of 120 MPa and a UTS of 230 MPa, along with an EL rate exceeding 10%. C611, developed by Alcoa for large die-cast components, exhibits excellent high-temperature fluidity and good demolding ability, with a UTS, YS and EL of 268 MPa, 123 MPa, and 16.2%, respectively. Additionally, Magna’s Aural 6 and Aural 6^+^ alloys share a composition similar to C611 but replace costly Mo and Zr with more economical Ti, thereby reducing industrial application costs. JDA 1 is an Al-Si alloy developed independently by SJTU. A heat-treatable aluminum alloy with YS and UTS values in the ranges of 180–210 MPa and 260–360 MPa, respectively, and an EL of 6–10%. LDHM-02, developed by the Lichung Group, exhibits excellent die-casting performance, filling capability, as well as high-temperature corrosion resistance and thermal stability. HTDA, developed by Guangdong Hongtu, exhibits good die-casting processability, flowability, resistance to thermal cracking, and a low shrinkage tendency. As can be seen from the above, Al-Si series aluminum alloys, with good fluidity, high strength and temperature resistance, are widely used in automotive die-casting, providing crucial support for achieving automotive lightweightness and safety. Al-Mg die-casting alloys with high strength and toughness, as well as excellent corrosion resistance, are increasingly emerging as highly promising aluminum alloys for die-casting. C666F is an Al-Mg alloy developed by Alcoa Inc, which uses Mn and Mg as primary alloying elements for solid-solution strengthening. Magsimal 610 alloy (AlMg6Si2Mn), developed by Rheinmetall AG in Germany, achieves a YS of 160 MPa, a UTS of 210 MPa, and an EL of 7%, though its die-casting performance remains relatively limited.

**Figure 4:**
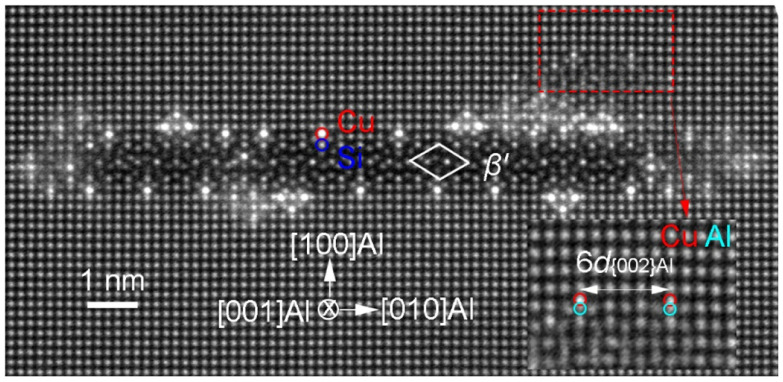
Mechanistic diagram of the action of Cu on the nucleation of precipitates [49].

The 5xxx series represents a class of non-heat-treatable aluminum alloys that achieve strength primarily through work hardening and can be softened by annealing. This contrasts sharply with heat-treatable alloys, which rely on solution treatment and aging for strengthening. The microstructure of non-heat-treatable aluminum alloys typically consists of α-Al grains and second-phase grains, which affect mechanical properties and are often refined through processes such as homogenization [46]. Heat-treatable precipitation-hardened forged aluminum alloys are highly valued in the aerospace industry [47]. However, when these aluminum alloys are exposed to high temperatures close to their aging treatment range, the nanoscale precipitates rapidly coarsen, a phenomenon commonly known as over-aging [48]. Numerous studies have examined the behavior of heat-treated aluminum alloys. For example, Zhu et al. [49] investigated the precipitation hardening and thermal stability of traditional non-heat-treatable AAxxx aluminum alloys, revealing that a small amount of Cu addition plays a critical role in AA3106 alloys. Cu promotes the nucleation of precipitates and facilitates the formation of β’ phase during aging. This increases the separation work at grain boundaries and reduces interface energy, thereby enhancing both the strengthening effect and thermal stability of the precipitates (Figure 4).

Zhang et al. [50] designed Al-Fe-Ni alloys using the CALPHAD method and they found that as the Ni decreased and the Fe increased, the main second phase changed from Al3Ni to Al10FeNi. This abundant fibrous rod-shaped Al10FeNi eutectic structure triggered a second-phase strengthening effect and promoted more uniform plastic deformation in the eutectic structure, thereby improving both strength and ductility. Hu et al. [51] investigated the effect of TiB_2_ particles on the non-heat-treated HPDC Al9Si0.6Mn alloy. In Figure 6 of the paper by Hu et al., with increasing TiB_2_ content, α-Al grains were refined and the morphology of eutectic Si was improved. Additionally, in Figure 4 of the paper by Hu et al., it was observed that in HPDC, α-Al formed coarse α_1_-Al and fine α_2_-Al grains, consistent with the reported bimodal α-Al microstructure in HPDC [52,53]. Both the YS and UTS of the alloy increase with the addition of TiB_2_ particles, while the EL decreases.

The 6xxx and 7xxx series aluminum alloys achieved excellent performance through heat treatment, but this process is energy-intensive and often causes issues such as part deformation. Microalloying and nanoparticle strengthening technologies have led to the development of an increasing number of high-performance aluminum alloys that require no heat treatment. Research on die-cast aluminum alloys has advanced from laboratory to industrial applications. Moving forward, efforts should focus on overcoming cost and performance bottlenecks, combining advanced process design, and further expanding their application in green manufacturing.

## 3. Development of Integrated Die-Cast Technology for New Energy Vehicles

New energy integrated die-casting aluminum alloy technology represents a key direction in automotive lightweight and manufacturing innovation [54,55]. This technology integrates multiple aluminum alloy parts into a single high-pressure casting, significantly reducing the number of parts and welding steps, improving body stiffness and production efficiency, while reducing energy consumption and cost. At present, Tesla has pioneered the use of 6000–7000 tons of die-casting machines to produce the rear bottom plate of the Model Y [56]. In the future, with advances in 10,000-ton die-casting equipment and new alloys, the technology is expected to extend to full-body manufacturing, promoting further lightweighting and cost reduction in new energy vehicles.

### 3.1. Integrated Die-Casting Technology

Integrated die-casting is now widely regarded as a revolutionary innovation that is fundamentally transforming automotive manufacturing. This process uses large die-casting machines to consolidate multiple separate parts into single large aluminum castings [57], replacing traditional connection methods such as stamping, welding, and assembly. Owing to its weight- and cost-saving efficiencies, integrated die-casting has become increasingly pivotal for lightweighting in new energy vehicle manufacturing [58,59]. In 2019, Tesla introduced IDCT, which highly integrates multiple separate parts and uses large-tonnage die-casting machines for one-time die-casting molding. This technology has sparked a manufacturing revolution in the automotive industry in terms of part performance, production efficiency, and manufacturing costs, while also providing a new path for new energy vehicle lightweighting. Integrated die-casting can effectively enhance the strength of local mounting points (seats and seatbelt anchor points), improve body performance (strength and stiffness), and optimize the lightweight coefficients. For example, Silafont-36 (AlSi10MnMg), developed by the German company Rhein, employs a high silicon content to ensure good die-casting fluidity and low thermal shrinkage; keeping the iron content low reduces mold adhesion issues. However, as integrated die-cast parts become more integrated and larger in size, it is no longer feasible to enhance product performance through conventional solution treatment followed by aging. As a result, developing aluminum alloys that require no heat treatment has become an inevitable choice for large thin-walled die-casting. Such materials must achieve mechanical properties comparable to those obtained through heat treatment in their as-cast or naturally aged states, primarily through microalloying and nanoparticle additions. Technological progress in integrated die-casting is driven by breakthroughs in die-cast aluminum alloys, sophisticated mold design and process optimization, specialized methods for large-scale complex castings, and high-fidelity property simulation [60].

### 3.2. Current Applications of Integrated Die-Casting

**Figure 5:**
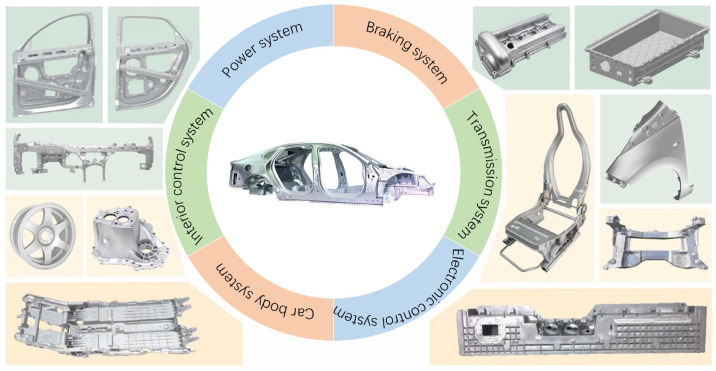
Integrated die-casting aluminum alloys for automotive components.

With the development of trends toward automotive electrification, energy efficiency, and environmental protection, research and application of integrated die-cast aluminum alloys have become increasingly important. These alloys are now widely adopted in automotive components (Figure 5). Currently, the dominant alloys for integrated die-casting are the Al-Si and Al-Mg alloys. Al-Si alloys are further divided into Al-Si-Mg types, such asSilafont-36 and Castasil-37. In Al-Mg alloys, Mg serves as the primary strengthening element, primarily acting as a solid-solution strengthener. Since Tesla first introduced the concept of integrated die-casting in 2019 and achieved significant weight reduction effects, numerous automotive companies have followed Tesla’s lead in developing IDC technologies. Table 1 lists vehicle models and corresponding castings that utilize die-cast aluminum alloys. As the foundational material for IDC, Al-Si series alloys are the focus of ongoing research aimed at designing and optimizing non-heat-treated aluminum alloy compositions, with an emphasis on cost-effective development. The priority is given to selecting lower-cost alloying elements to gradually reduce the overall manufacturing costs.

The overarching objective is to promote the overall performance of aluminum alloys by elucidating the relationships between microstructure and phase transformation and by optimizing alloy composition and heat treatment schedules to simultaneously enhance both strength and toughness. Given the advantages of Al-Mg alloys, future research should focus on composition optimization to overcome their casting performance limitations, making them an ideal material for next-generation automotive components. A comparative analysis of international and domestic studies on high-strength, high-toughness aluminum alloys reveals clear composition–property relationships. Die-cast aluminum alloys, due to their unique advantages, demonstrate significant application potential in die-casting. Although these alloys originated in new energy vehicles, they are not limited to this technology; traditional vehicles can also achieve lightweightness by replacing steel with aluminum. Research has shown that die-cast aluminum alloys not only exhibit excellent as-cast properties, such as high toughness and good elongation, but can also further enhance strength and thermal stability through precise chemical composition design. Table 3 shows the integrated die-cast parts used by selected automakers in their respective models.

## 4. Influence of Alloying Elements on the Al-Si-Mg and Al-Mg-Si Series Aluminum Alloys

An emerging trend in automobile manufacturing is the replacement of steel welded and stamped body structures with one-piece aluminum giant castings produced by high-pressure die-casting technology [61]. Currently, alloys for IDC parts can be divided into Al-Si and Al-Mg series [61,62]. Microalloying constitutes a primary approach for enhancing the casting characteristics of these aluminum alloys, and research confirms that Al3M intermetallic phases serve as effective modifiers [63]. The incorporation of different alloying elements induces distinct alterations in the morphology and distribution of precipitates, which governs the alloy’s macroscopic behavior. Consequently, researchers have focused on elements such as Cu, Zn, and rare earths metals to achieve specific microstructural and property improvements.

### 4.1. The Effect of Alloying Elements on Al-Si-Mg Series Alloys

Al-Si-Mg alloys represent important materials in lightweight automotive die-casting, offering a well-balanced set of properties for structural components [64,65,66]. Microalloying is one of the most effective methods for improving the performance of aluminum alloys. Different alloying elements influence precipitated phases in specific ways, governing their size and morphology to distinctly alter an alloy’s final microstructure and properties.

#### 4.1.1. The Effect of Cu on Al-Si-Mg Series Alloys

**Figure 6:**
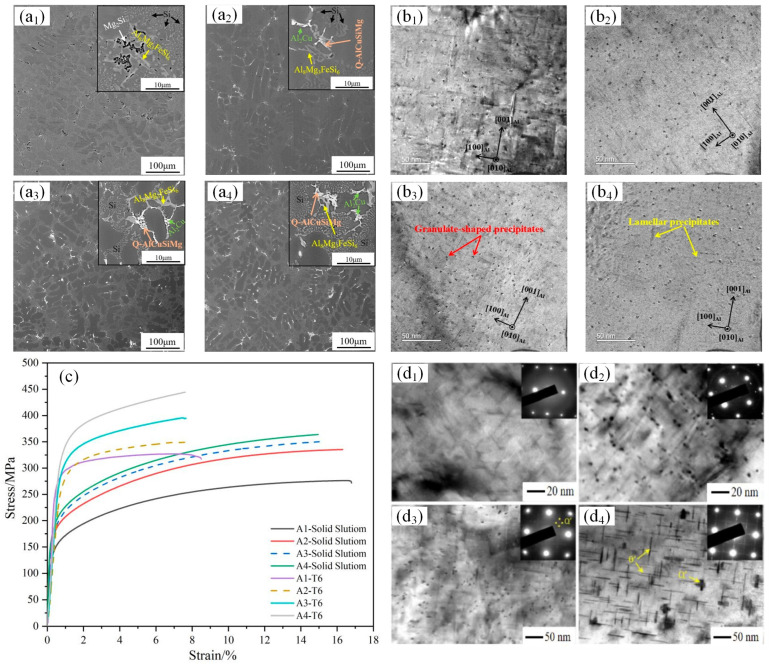
SEM-BSE images of as-cast alloys: (**a_1_**) 0.01Cu, (**a_2_**) 0.89Cu (**a_3_**) 1.43Cu, and (**a_4_**) 2.08Cu. Bright-field TEM images of the alloys after aging treatment: (**b_1_**) 0.01Cu, (**b_2_**) 0.89Cu (**b_3_**) 1.43Cu, (**b_4_**) and 2.08Cu. (**c**) Engineering stress–strain curves of the alloys after solid-solution and aging treatment [69]. Bright-filed TEM images with corresponding SAED patterns of aged alloy: (**d_1_**) the precursor of the β″ phase; (**d_2_**) β′ phase; (**d_3_**) Q′ phase; (**d_4_**) Q′ and θ′ phase. TEM—Transmission Electron Microscopy; SAED—Selected-Area Electron Diffraction [70].

Introducing additional precipitation stages or altering the precipitation sequence can be an effective strategy for enhancing alloy strength. In aluminum alloys, Cu serves as an effective age-hardening element by forming Al_2_Cu precipitates [67]. Al-Si-Mg alloys undergo aging from a supersaturated solid solution (SSS), following the precipitation sequence [68]: SSS → GP zones → β″ (Mg_5_Si_6_) → β′ → β (Mg_2_Si). Solution and aging treatment effectively enhance tensile strength, but they inevitably sacrifice ductility. Zhou et al. [69] investigated the microstructural evolution and mechanical properties of Al-Si-Mg alloys with varying Cu contents. Figure 6(a_1_–a_4_) shows SEM images of the alloys. In the absence of Cu, the second phase is Mg_2_Si; when Cu is added, Al_2_Cu appears as the second phase, and the content of Al_2_Cu increases with increasing Cu content. Figure 6(b_1_–b_4_) shows the TEM microstructure of the alloy. It can be observed that during the failure process, the number density of nanoscale precipitation phases increases with increasing Cu content. Figure 6c shows the engineering stress–strain curves of the alloys after solution and aging treatments. In both the solution-treated and aged states, the mechanical properties increase with increasing Cu content. The variation in alloy strength at different copper contents can be attributed to both solution strengthening and aging strengthening. In the solution-treated specimens, an increase in copper content leads to a higher solute concentration, resulting in a stronger solution-strengthening effect. After aging treatment, the mechanical properties of the alloy improved significantly; in Al-Si-Mg-xCu casting alloys, the effect of aging treatment on strength was more pronounced than that of solution treatment. Furthermore, this indicates that the elongation of the aged specimens decreased, which was due to the inverse relationship between strength and ductility. Zhang et al. [70] investigated the effect of the Cu/Mg ratio on precipitation phases in Al-Si-Mg-Cu alloys. As shown in Figure 6(d_1_–d_4_), it was found that when the copper content was low (1.04 wt.% Cu) and the Cu/Mg ratio was 2, the alloy tended to form a precursor to the β′ phase; when the copper content was 1.06 wt.% and the Cu/Mg ratio was 1, the alloy tended to form the β′ phase; when the copper content was 2.08 wt.% and the Cu/Mg ratio was 4, the alloy tended to form the Q′ phase; however, when the copper content reached 3.98 wt.% and the Cu/Mg ratio remained at 4, the alloy contained the θ′ phase in addition to a small amount of Q′. Zhang et al. [71] demonstrated that increased Cu content promotes work hardening, increasing ultimate tensile strength. Increasing the Cu content to 3% increased the YS to 266 MPa and the UTS to 660 MPa, but reduced the EL to 16.68%. Overall, Cu addition contributes to a favorable strength–ductility balance compared to other alloying approaches.

Xue et al. [71] studied the precipitation behavior of the Al-8Si-0.6Mg alloy at peak aging and identified the β″ phase as the primary strengthening precipitates [72]. Conversely, the peak-aged Al-8Si-2Cu-0.6Mg alloy formed Q′ precipitates because its Cu content (>1.25%) inhibits β″ formation and shifts precipitation towards θ′ or a θ′/Q′ mixture [73]. Further research confirms that the primary strengthening phase in peak-aged Al-1.13Si-0.6Cu-0.67Mg alloys is the QP phase, which acts as a precursor to Q′. As shown in in Figure 4 of reference [14], the needle-like or rod-like precipitates in Figure 4a of reference [14] are consistent with the previously described phase and those in Figure 4i of reference [14] are identified as QP phase. Addition of 2% Cu to the Al-8Si-0.6Mg base alloy accelerates precipitation kinetics, thereby imparting a greater age-hardening response and enhanced thermal stability. Theoretical analysis further indicates that although QP precipitation is favored over β phase, its growth is limited by the low diffusivity of Cu. The stress–strain curves of the Al-8Si-0.6Mg and Al-8Si-2Cu-0.6Mg alloys are compared in Figure 5 of reference [14]. In the as-cast condition, the Al-8Si-2Cu-0.6Mg alloy shows a slightly higher yield and slightly higher tensile strengths than the Al-8Si-0.6Mg alloy, with a slight reduction in elongation. Notably, after peak aging, the Cu-containing alloy achieves an excellent balance between strength and ductility.

Compared with gravity or die-casting, thixotropic casting offers superior cooling rates, which improve the fluidity of alloy elements within the master alloy. This allows cast aluminum alloys that have not undergone solution treatment to still exhibit pronounced age-hardening properties. Through Cu content optimization, Yamamoto et al. [74] fabricated a high-performance, T6-treated Al-7Si-0.6Mg alloy via thixotropic casting. The results indicate that increasing the Cu content to 0.6% increases both YS and UTS. Further Cu addition significantly reduces the EL of alloys. The enhanced strength resulting from Cu addition is due to a transformation of the primary strengthening precipitates from the shearable β″ phase to the non-shearable θ′ phase, the latter offering greater resistance to dislocation motion. In summary, the introduction of Cu can alter the precipitation sequence during aging to raise the alloy’s strength and promote work hardening, thereby enhancing the alloy’s UTS. However, excessive Cu can induce pronounced segregation, consequently impairing alloy performance. Therefore, the Cu content must be carefully optimized to achieve a superior combination of properties.

#### 4.1.2. The Effect of Zn on Al-Si-Mg Series Alloys

Zn is one of the most prevalent alloying elements added to Al-Si-Mg alloys and acts as a key modifier. Its addition can modify the precipitation behavior and mechanical properties, but the resultant outcome depends strongly on the Zn content and its synergistic effects with other alloying elements. Adding Zn to Al-Si-Mg alloys promotes the formation of MgZn_2_, a high-strength precipitation phase that significantly enhances age-hardening. Hu et al. [75] specifically studied the influence of Zn on the Al-8Si-0.6Mg-0.1Cu alloy. They observed that the addition of Zn accelerated strengthening, and higher Zn contents led to markedly improved strength and elongation.

During HPDC, ESC nucleates at the die–melt interface. The disparity in cooling rates from surface to center, together with the presence of ESCs, results in significant microstructural heterogeneity. Jiang et al. [76] identified 2 wt.% Zn as a critical threshold and found that raising the Zn content increased undercooling and the α-Al nucleation rate. At 2 wt.% Zn, the ESCs within the segregation zone became refined and more uniform, despite the width of the zone remaining unchanged. Mechanically, 1 wt.% Zn shows little effect, but 2 wt.% Zn produces significant improvements in YS, UTS, and EL. In summary, the addition of Zn promotes the formation of the high-strength MgZn_2_ precipitate, which enhances the alloy’s strength [77,78,79]. It also increases undercooling, accelerates the nucleation of α-Al, and thereby promotes microstructural refinement and homogeneity. This refinement mechanism, coupled with Zn’s role as a strengthening element, can substantially enhance alloy performance. Nevertheless, the Zn content must be carefully optimized to achieve a synergistic balance in Al-Si-Mg alloys.

#### 4.1.3. The Effect of Other Alloying Elements on Al-Si-Mg Series Alloys

In addition to the elements previously discussed, Ti and Zr also profoundly influence the microstructure and properties of aluminum alloys. Wang et al. [80] demonstrated that adding Ti and Zr to an Al-7Si-0.6Mg alloy promoted the formation of L12-(Al, Si)3(Ti, Zr) phases, which enhanced mechanical performance.

**Figure 7:**
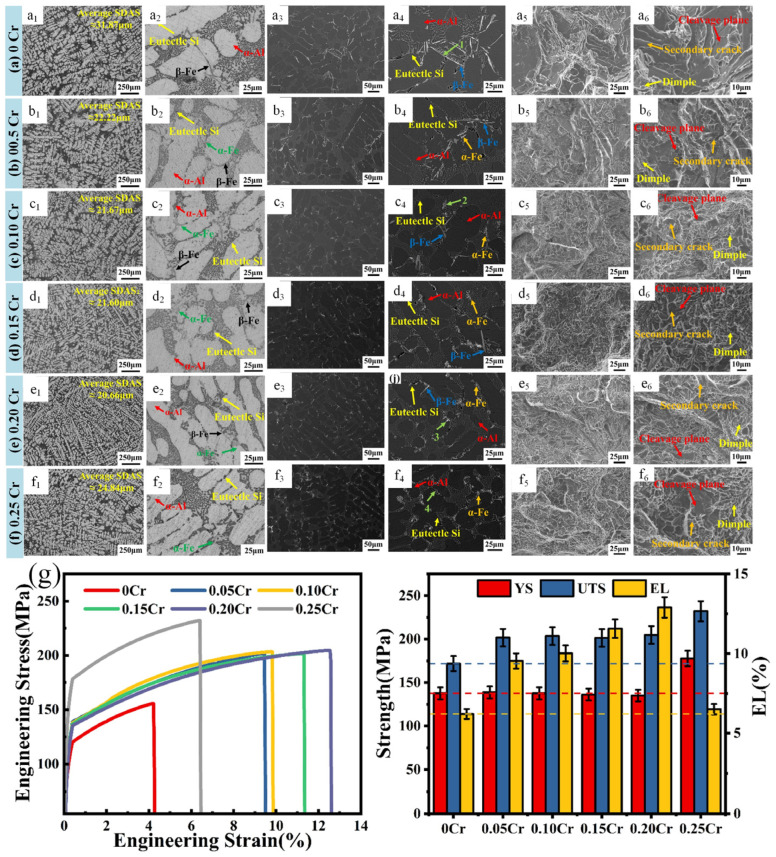
Optical microstructures/SEM/fracture morphology of Al-7Si-0.35Mg-0.35Fe alloys with different Cr contents: (**a_1_**–**a_6_**) 0Cr; (**b_1_**–**b_6_**) 0.05Cr; (**c_1_**–**c_6_**) 0.10Cr; (**d_1_**–**d_6_**) 0.15Cr; (**e_1_**–**e_6_**) 0.20Cr; (**f_1_**–**f_6_**) 0.25Cr. (**g**) Mechanical properties of Al-7Si-0.35Mg-0.35Fe alloys [87].

In contrast, iron exerts a detrimental effect on aluminum alloys. Its presence deteriorates elongation, fatigue properties, and often corrosion resistance [81,82,83,84]. The harmful effects of Fe stem from the precipitation of iron-rich intermetallic particles [85,86]. Le et al. [87] improved the properties of the Al-7Si-0.35Mg alloy by modifying the iron-rich phase through the addition of Cr. As shown in Figure 7a–f, the optical microstructure, SEM microstructure, and fracture microstructure of Al-7Si-0.35Mg-0.35Fe alloys with different chromium contents are presented. As the Cr content increases, the secondary dendrite arm spacing (SDAS) initially decreases and then increases. Meanwhile, the addition of chromium has a profound effect on the morphology and distribution of the iron-rich phase. In the 0Cr alloy, numerous needle-like β-Fe phases are present. As the chromium content increases, the iron-rich phase gradually transforms from the β-Fe phase to the α-Fe phase. In the 0.05–0.20 wt.% chromium range, the number of β-Fe phases continues to decrease, gradually being replaced by finer and more regularly shaped α-Fe phases. Figure 7g shows the engineering stress–strain curves and corresponding tensile properties of Al-7Si-0.35Mg-0.35Fe alloys with different chromium contents. All chromium-containing alloys exhibit superior mechanical properties compared to the 0Cr alloy. As the chromium content increases within the range of 0–0.20 wt.%, the elongation (EL) of the alloy increases significantly, while the ultimate tensile strength (UTS) and yield strength (YS) remain nearly constant. However, when the chromium content is increased to 0.25 wt.%, the elongation decreases significantly, while both the UTS and YS increase significantly. Among the alloys studied, the 0.20Cr alloy achieves the optimal balance between strength and ductility. The figure shows the fracture morphology of Al-7Si-0.35Mg-0.35Fe alloys with different chromium contents. The fracture surface of the 0Cr alloy exhibits large cleavage planes and microcracks, demonstrating brittle fracture characteristics. After chromium addition, the fracture surface primarily consists of small cleavage planes, microcracks, and indentations. Increasing the chromium content significantly reduces the number and size of cleavage planes while increasing the number of indentations and refining their size. The morphology of the iron-rich phase changes from acicular to rod-like and block-like. The addition of chromium significantly reduces potential crack nucleation sites, thereby enhancing the alloy’s plastic deformation capacity. Liu et al. [88] systematically investigated the effect of iron content in the A366-T6 aluminum alloy. Figure 1 of the paper by Liu et al. shows that a higher Fe content results in more numerous and larger Fe-rich IMPs, which adopt a coarse needle-like morphology. The room-temperature tensile curves indicate that the properties of the alloy gradually decrease with increasing Fe content. Moreover, a higher Fe content in the matrix intensifies corrosion, shifting the corrosion behavior from filiform to pitting, with larger and deeper pits. Therefore, it is particularly important to minimize the Fe content during casting. Mn acts as a neutralizing agent in Fe-containing aluminum alloys, converting β-Al6FeSi into α-Al (Mn, Fe) Si. As a non-shearable precipitate, it promotes dislocation cross-slip and contributes significantly to alloy strengthening. Kim et al. [89] reported that the synergistic addition of Mn and Cr is more effective in modifying β-Al6FeSi than Mn alone. Ni also acts as an iron neutralizer, transforming brittle plate-like β-Al6FeSi into elongated Al9FeNi and Al3Ni phases. Researchers have developed a new engine application alloy based on Al-Si-Mg-Cu-Fe-Ni that exhibits excellent high-temperature and fatigue properties. Casari et al. [90] studied the effects of Ni and V additions to A366 aluminum alloys and observed the aforementioned Ni-rich phases in both as-cast and T6 conditions. Their results reveal the beneficial influence of Ni and V additions on the performance of A366 aluminum alloys.

The microalloying element Sn exhibits high diffusivity and a strong affinity for vacancy in aluminum alloys. Huang et al. [91] investigated a high-Sn A366 alloy and found that Sn addition markedly refines α-Al dendrites and modifies eutectic Si morphology. In summary, elements such as Ti, Zr, Cr, and Sn improve alloy performance through the formation of specific phases. In contrast, Fe adversely affects mechanical properties and corrosion resistance, underscoring the importance of minimizing its content in casting alloys.

#### 4.1.4. The Effect of Rare Earth Elements on Al-Si-Mg

**Figure 8:**
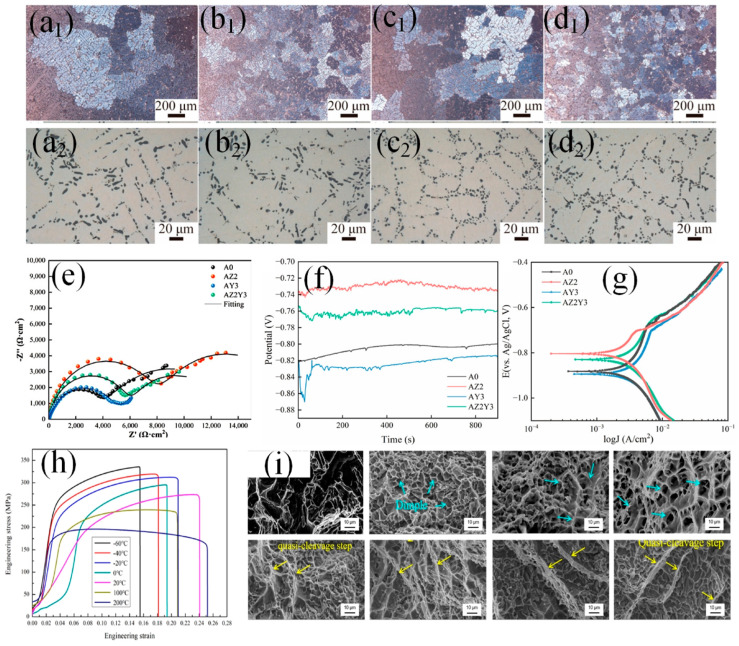
OM micrographs of four alloys: (**a_1_**,**a_2_**) Al-Si-Mg, (**b_1_**,**b_2_**) Al-Si-Mg-0.2Zr, (**c_1_**,**c_2_**) Al-Si-Mg-0.3Y, and (**d_1_**,**d_2_**) Al-Si-Mg-0.2Zr-0.3Y. (**e**) Nyquist plots. (**f**) OCP curve. (**g**) Potentiodynamic polarization curve [93]. (**h**) Engineering stress–strain curves of the alloy at different temperatures. (**i**) Fracture morphologies of the untreated alloy and Sc-Sr composite modified alloy under different temperatures [94].

Rare earth elements have special physical and chemical properties, and they have demonstrated excellent refining and modification effects in Al-Si-Mg alloys [92,93,94]. In recent years, cost-effective La has attracted considerable attention for its dual role in both eutectic modification and grain refinement in 4xxx aluminum alloys [93,95]. Researchers are exploring the potential of rare earth elements to improve the performance of Al-Si-Mg series alloys. For example, focusing on Al-Si-Mg alloys, Ye et al. [93] investigated the effects of Zr and Y on the corrosion behavior of T6-heat-treated Al-Si-Mg alloys. As shown in Figure 8(a_1_–d_2_), the addition of Zr and/or Y altered the sizes of the primary α-Al dendrites and eutectic silicon particles. In the A0 alloy, the α-Al grains exhibit a coarse dendritic structure, while the eutectic silicon particles are partially spherical and rod-shaped. The addition of 0.2 wt% Zr to the A356 alloy significantly refines the α-Al grains, transforming them from coarse particles into smaller spherical ones. Figure 8e shows the impedance spectrum of the alloy. As can be seen from the figure, the AZ2 alloy has a larger arc radius. Figure 8g shows the polarization curves of the alloys. The AZ2 alloy exhibits a higher self-corrosion potential and a lower corrosion current density, indicating that the AZ2 alloy is less prone to corrosion and has a slower corrosion rate. This is because the introduction of Y into the Al–Si–Mg alloy promotes the formation of a thicker and more uniform passive film. Furthermore, adding 0.3 wt.% Y to the Al–Si–Mg alloy increases the density of eutectic silicon particles, leading to a more extensive network of intergranular corrosion pathways and more electrical connections, thereby reducing corrosion resistance. It is well known that the mechanical properties of Al–Si–Mg alloys are greatly affected by temperature, which is detrimental to their application. Previous studies have shown that, due to its stability at high temperatures, Al3Sc is considered a key element in the development of high-performance aluminum-based alloys. Zhang et al. [94] investigated the tensile properties of the Al-7.12Si-0.36Mg-0.2Sc-0.005Sr alloy at different temperatures (Figure 8h), demonstrating that the alloy exhibits excellent yield strength stability over the temperature range of 20–200 °C. Figure 8i shows the representative microstructures of fracture cross-sections for the untreated alloy and the Sc-Sr composite-modified alloy at different temperatures. From the figure, we can observe the presence of needle-like and block-like eutectic silicon in the untreated Al-Si-Mg alloy. However, most of the eutectic silicon in the Sc-Sr composite-modified alloy appears spherical in cross-sections at different temperatures. Strontium is primarily distributed at the growth front of the eutectic silicon to promote its isotropic growth, ultimately transforming the morphology of the eutectic silicon from needle-like to short fibers or fine grains. At a high temperature of 200 °C, the predominantly dispersed Al_3_Sc phase hinders dislocation motion during subsequent plastic deformation, leading to dislocation entanglement and accumulation. The high-density dislocation network forms a deformation substructure, and the interaction between this substructure and Si nanoparticles results in matrix strengthening. Xu et al. [96] studied the impact of Sc addition and observed a pronounced microstructural refinement in the F367 alloy. The grain refinement mechanism involves a eutectic reaction at approximately 600 °C that forms Al_3_Sc particles [97]. These particles have lattice parameters similar to α-Al and act as potent heterogeneous nucleation sites. Consequently, adding 0.8 wt.% Sc to the as-cast F367 alloy increases its YS, UTS, and EL. Cai et al. [93] reported that up to 1.0 wt.% La, the eutectic Si particles in the A366 alloy remain similar to those in the unmodified A366 alloy. At 1.0 wt.% La, a fully modified fine-fiber eutectic structure appears. Furthermore, thermal analysis indicates that La addition lowers both the eutectic nucleation and growth temperatures. In terms of mechanical properties, while UTS shows no significant variation with La content, EL is improved specifically at 0.6 wt.% La. Studies show that the combined addition of La and grain refiners can further enhance the mechanical properties of Al-Si-Mg alloys. However, grain refiners commonly used in aluminum alloys may be “poisoned” by Si in Al-Si alloys. Zr is inexpensive and can significantly refine α-Al and is more compatible with La. Cao et al. [98] found that in the Al-7Si-0.6Mg alloy, combined La-Zr addition refines grains more effectively than individual addition. In addition, combined addition of La and Zr simultaneously improves tensile strength and elongation.

Previous studies indicate that adding small amounts of Ce (0.06–1%) to Al-Si alloys produces several effects, most notably refining eutectic Si into a fibrous morphology. In A366 Al-Si-Mg alloy, 1% Ce was found to significantly improve both microstructural modification and mechanical properties. A key finding was that ductility increased markedly yet ultimate tensile strength did not, which was attributed to the precipitation of the Al-17Ce-12Ti-2Si intermetallic compound. Cai et al. [99] studied the influence of La and Ce on A366 alloys and observed that both elements refined the needle-like eutectic Si into finer, more spherical particles. To quantify this modification, the average area and aspect ratio of the Si particles were measured via quantitative metallography using an IMAQ Vision Builder 6 image analyzer, calculated as follows:

(1) $$Meanarea=\frac{1}{m}\sum_{j=1}^{m}\;{\left(\frac{1}{n}\sum_{i=1}^{n}\;A_{i}\right)}_{j}$$

(2) $$\mathrm{Aspect}\;\mathrm{ratio}=\frac{1}{m}\sum_{j=1}^{m}\;{\left[\frac{1}{n}\sum_{i=1}^{n}\;{\left(\frac{L_{i}}{L_{s}}\right)}_{i}\right]}_{j}$$

where “A_i_” is the measured area of an individual silicon particle, “L_i_/L_s_” is defined as the ratio of the longest dimension to the shortest dimension, “n” is the number of particles counted within a single micrograph, and “m” is the total number of micrographic fields analyzed. The calculation results indicate that, compared to La, Ce-modified alloys exhibit larger average areas and aspect ratios, meaning that the modification efficiency of 1 wt.% La is higher than that of 1 wt.% Ce. For example, EL increased from 2.8% in the unmodified alloy to 6.9% in the La-modified alloy, which was higher than the 6.6% in Ce-modified alloy.

Shi et al. [100] found that adding Er to A366 alloys refined both α-Al grains and eutectic Si. This refinement had little effect on as-cast properties, but after T6 treatment it led to a marked improvement in overall alloy performance. Tang et al. [101] investigated the influences of Nd on the Al-7Si-0.3Mg-0.3Fe alloy. As shown in Figure 7 of the paper by Tang et al., the size of Nd-rich particles increases with higher Nd contents, and the particles exhibit distinct morphologies at different Nd contents. Furthermore, the study also found that while Nd addition did not markedly alter the content of the β-AlFeSi phase, it induced a substantial increase in the fraction of the Π-AlSiMgFe phase. In the as-cast condition, Nd addition did not markedly alter the alloy’s UTS. After T6 treatment, however, the UTS of the alloy increased significantly, which is consistent with the observations of Shi et al., indicating that Er and Nd have similar effects on Al-Si-Mg alloys. The application of Er- and Nd-modified Al-Si-Mg series aluminum alloys remains a considerable challenge. In summary, rare earth elements play significant yet distinct roles, with different elements producing various strengthening effects.

### 4.2. The Influence of Alloying Elements on Al-Mg-Si Series Alloys

Due to their outstanding specific strength and castability, Al-Mg-Si alloys are extensively used in automotive lightweight structures, making strength enhancement a priority. One effective approach is raising the Si content at a fixed Mg level to promote the precipitation of strengthening β″ (Mg_5_Si_6_) phases. Al-Mg alloys, on the other hand, offer a lower density and superior corrosion resistance, with strength generally increasing with Mg content. More broadly, alloy strength is predominantly governed by precipitation effects relative to the base matrix. Current strategies for controlling precipitates involve adding specific compounds or rare earths to modify precipitate–matrix interactions. This is particularly relevant for age-hardenable Al-Mg-Si alloys, whose microstructure and mechanical properties optimized through heat treatment have been widely studied in recent years. Their precipitation sequence exhibits considerable complexity. The broadly accepted progression is [102]: SSS → GP zone → β″ (Mg_5_Si_6_) → β′ → β (Mg_2_Si). Here, GP zones are spherical, β″ is needle-like, and β′ is rod- or plate-shaped. Because of its poor coherency with the matrix, its strengthening effect is not as good as that of β″. For Cu-containing Al-Mg-Si alloys, the broadly accepted progression is [103]: SSS→ GP region (atomic cluster of former β″) → β″ + L + QP + QC → β′ + Q′ + θ′ → Q + θ. Recent studies indicate that incorporating cost-effective modifiers can transform the microstructure of complex Mg_2_Si eutectics, offering a viable route to enhance alloy performance [104]. Separately, it has also been found that both strength and ductility improve with Cr content up to 0.2 wt.%, beyond which they decline. Excessive Cr reacts with Fe and Si to form coarse AlFeCrSi phases, which consume considerable amounts of Si and thereby reduce the precipitation of Mg_2_Si strengthening phases [105].

#### 4.2.1. The Effect of Cu on Al-Mg-Si Series Alloys

**Figure 9:**
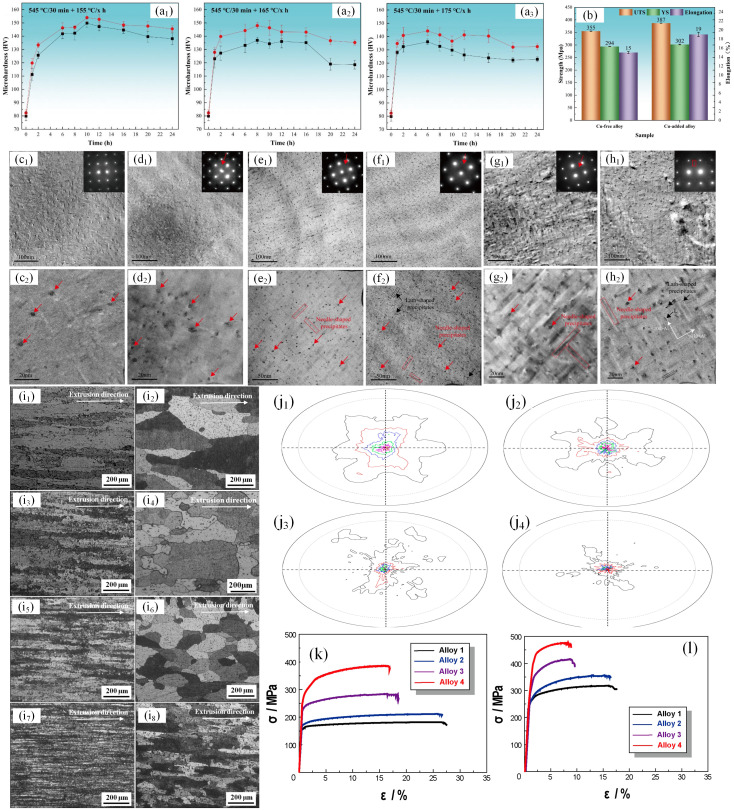
The age-hardening curves of Cu-free and Cu-added alloys at various temperatures: (**a_1_**) aging at 155 °C, (**a_2_**) aging at 165 °C, (**a_3_**) and aging at 175 °C. Room-temperature tensile mechanical properties of Cu-free and Cu-containing alloys following various aging treatments. (**b**) Peak-aged condition (155 °C for 10 h). TEM images of two alloys in the under-aged condition (155 °C for 2 h): (**c_1_**,**c_2_**) Cu-free alloy; (**d_1_**,**d_2_**) Cu-added alloy. TEM bright-field images (**a**–**d**) and the corresponding precipitate length distributions for two alloys in the peak-aged condition (155 °C for 10 h): (**e_1_**,**e_2_**) Cu-free alloy; (**f_1_**,**f_2_**) Cu-added alloy. TEM images of two alloys in the over-aged condition (155 °C for 24 h): (**g_1_**,**g_2_**) Cu-free alloy; (**h_1_**,**h_2_**) Cu-added alloy [106]. Optical micrographs of alloys at different states: (**i_1_**) as-extruded 0.5Cu, (**i_2_**) as-quenched 0.5Cu, (**i_3_**) as-extruded 1.0Cu, (**i_4_**) as-quenched 1.0Cu, (**i_5_**) as-extruded 2.5Cu, (**i_6_**) as-quenched 2.5Cu, (**i_7_**) as-extruded 4.5Cu, and (**i_8_**) as-quenched 4.5Cu. The {200} pole figures of four as-quenched alloys: (**j_1_**) 0.5Cu 1, (**j_2_**) 1.0Cu, (**j_3_**) 2.5Cu, and (**j_4_**) 4.5Cu. Tensile curves of the alloys in different heat treatment states: (**k**) as-quenched and (**l**) peak-aged [107].

Cu is a pivotal microalloying element in Al-Mg-Si alloys, strengthening the material through solid-solution hardening and the formation of precipitation phases. Its presence refines precipitate distribution and enhances the age-hardening response [106,107]. Furthermore, Cu addition can mitigate the negative impact of natural aging. Zhao et al. [106] investigated the effects of copper on the precipitation behavior and mechanical properties of Al-Mg-Si alloys. The results showed that the addition of copper significantly enhanced the alloy’s age-hardening response (Figure 9(a_1_–a_3_)), increased hardness and room-temperature tensile strength at the peak age-hardening state (Figure 9b), and reduced the softening rate during over-aging. The peak age-hardening tensile strength of the copper-added alloy (387 MPa) was approximately 9% higher than that of the copper-free alloy (355 MPa), and the elongation at break of the copper-added alloy reached 19%, far exceeding the 15% of the copper-free alloy. Copper promotes the precipitation of the unaged and peak-aged β “strengthening phase” in the alloy grains (Figure 9(c_1_–d_2_)), while simultaneously promoting the formation of strip-like Q’ and L phases in the microstructures of the peak-aged (Figure 9(e_1_–f_2_)) and over-aged (Figure 9(g_1_–h_2_)) states. This enhances the room-temperature tensile properties of the alloy in the peak-aged state and reduces the degradation of properties during over-aging. Chen et al. [107] investigated the effect of Cu content on the microstructure and strengthening mechanisms of Al-Mg-Si alloys. As the Cu content increased, the grain size gradually decreased (Figure 9(i_1_–i_8_)), and they confirmed that recrystallization was more pronounced in alloys with higher copper contents, since the {200} fibrous texture is a typical recrystallization texture in aluminum alloys (Figure 9 (j_1_–j_4_)). Figure 9k,l show the tensile curves of the alloy in different conditions; as the Cu content increases, the strength of the alloy increases. Wang et al. [108] investigated the influence of Cu content on the Al-6.6Mg-2Si alloy by presenting EBSD orientation maps, OM, and SEM images of the Al-6.6Mg-2Si-xCu alloys. They found that the Al-6.6Mg-2Si alloy exhibited a fibrous composite structure of primary α-Al and eutectic Al/Mg_2_Si, with equiaxed, randomly oriented α-Al grains. The addition of Cu exerted a strong growth restriction effect. This was evidenced by a grain size decrease from 36.6 ± 10.6 μm to 21.0 ± 6.6 μm with 0.26% Cu and a reduction in secondary dendrite arm spacing from 10 ± 2.2 μm to 6.8 ± 0.86 μm as Cu increased to 0.76 wt.%, illustrating Cu’s effectiveness in refining the as-cast microstructure. Engineering strain curves for the Al-6.6Mg-2Si-xCu alloy with corresponding statistical data were presented in Figure 9 of the paper by Liu et al. The base alloy exhibits moderate strength (YS: 126 MPa, UTS: 268 MPa) but excellent ductility (EL: 12.1%). Adding 0.06 wt.% and 0.1 wt.% Cu elevates the YS by 13 MPa and 110 MPa, respectively, while preserving ductility, resulting in an excellent strength–ductility synergy.

Cu enhances the thermal stability of Al-Mg-Si alloys. For instance, Jin et al. [109] found that introducing 0.6% Cu to a 6082 alloy significantly increased its peak hardness. Clustering, a critical reaction in age-hardenable alloys, significantly influences final properties and provides a pathway to an optimal strength–ductility balance. The incorporation of copper imparts a notable improvement in precipitation hardening, primarily by refining clusters and precipitates. This improvement is attributed to Cu’s ability to modify the energy landscape for cluster formation; it increases the system’s free energy, thereby lowering the driving force required for clustering and enhancing it during natural aging [110]. As Aster et al. [111] concluded, Cu’s mechanical property benefits in Al-Mg-Si alloys stem from this refinement, which increases the number and volume fraction of clusters and precipitates. Wu et al. [112] examined Cu-containing twin-roll cast Al-Mg-Si alloys and observed that Cu addition did not reduce centerline segregation. However, after cold rolling and solution treatment, segregation was almost fully dissolved in the alloys with 0.8% and 1.2% Cu, as shown in Figure 4 of the paper by Wu et al. In conclusion, copper addition refines microstructural and increases precipitate density, thereby synergistically enhancing mechanical strength. Additionally, it improves the thermal stability and helps alleviate centerline segregation encountered in twin-roll casting. However, excessive Cu can reduce the alloy’s corrosion resistance and potentially worsen segregation. Therefore, the Cu content must be carefully controlled during alloying to achieve a more comprehensive and superior performance.

#### 4.2.2. The Effect of Zn on Al-Mg-Si Series Alloys

**Figure 10:**
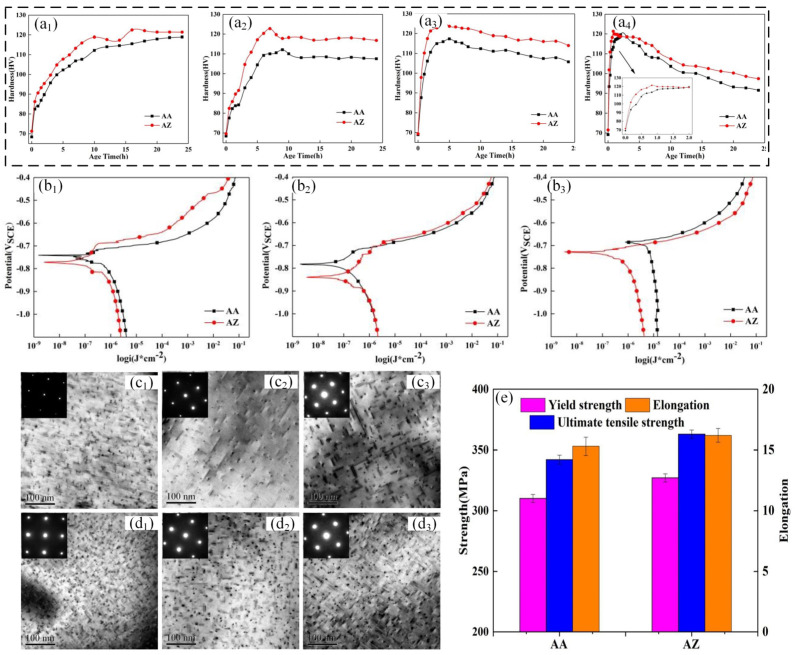
Age-hardening curves of the alloy with 0.05Zn and with 0.2Zn at different temperatures: (**a_1_**) 150 °C, (**a_2_**) 170 °C, (**a_3_**) 190 °C, and (**a_4_**) 210 °C. Potentiodynamic polarization curves of alloys with 0.05Zn and with 0.2Zn under different aging conditions at 170 °C: (**b_1_**) under-aged, (**b_2_**) peak-aged, and (**b_3_**) over-aged. TEM of intragranular precipitates of 0.05Zn alloy of needle phase under 170 °C aging: (**c_1_**) under-aged state, (**c_2_**) peak-aged state, and (**c_3_**) over-aged state. TEM of intragranular precipitates of 0.2Zn alloy of needle phase under 170 °C aging: (**d_1_**) under-aged state, (**d_2_**) peak-aged state, and (**d_3_**) over-aged state. (**e**) Tensile test results of 0.05Zn alloy and 0.2Zn alloy under peak-aged conditions at 170 °C [113].

The influence of Zn in Al-Mg-Si alloys has garnered increasing attention in recent years [113,114,115], yet its specific impact on mechanical properties remains ambiguous. Cai et al. [116] observed that Zn did not markedly increase the YS or reduce the EL in their alloy, though it raised the peak strength while extending the aging time needed to achieve it. In contrast, reference [117] points out that adding 3.61% Zn to an Al-10.76Mg-2.86Si-0.610Mn alloy increases the YS by 38% but reduces ductility by more than threefold. Similarly, Li et al. [118] found that adding 6.62% Zn to the Al-8.1Mg-2.6Si alloy increased the YS by 86%, accompanied by a more than threefold reduction in the EL. These results suggest that Zn addition does not lead to a simultaneous enhancement in strength and ductility in Al-Mg-Si alloys. Nevertheless, strengthening mechanisms in specific systems has been detailed. For instance, Chis et al. [119] attributed the strength increase in a 0.6 wt.% Zn-containing Al-Mg-Si-Cu alloy to the formation of GP(II) zones and η′ (Mg_2_Zn) precipitates. Although Zn addition generally promotes the formation of clusters or precipitates within the Al-Mg-Zn system, reference [119] reported that in an Al-Mg-Si-Cu alloy with 0.6 wt.% Zn, aging does not induce Zn-rich clusters or precipitates. Instead, Zn remains predominantly in solid solution, where it facilitates the enrichment of Mg, Si, and Cu in atomic clusters, GP zones, and β″ phases. Similarly, reference [120] suggested that Zn atoms uniformly aligned within atomic columns and exhibited only a limited influence on the β″ precipitates. According to [121], Zn was almost entirely incorporated into the grain-boundary Q phase rather than remaining in the matrix. The elevated Zn concentration in this phase lowers its corrosion potential compared to the matrix, leading to intergranular attack. Chi et al. [119] investigated the effects of small amounts of Zn on the precipitation behavior and corrosion resistance of Al-Mg-Si alloys. The alloy containing 0.2% Zn reached peak aging first (Figure 10(a_1_–a_4_)). After adding 0.2% Zn, the density of the precipitates was higher (Figure 10(c_1_–d_3_)). The figure shows the tensile test results of the alloys after peak aging at 170 °C; the alloy with 0.2% Zn exhibited better performance. Zn also affects the corrosion resistance of Al-Mg-Si alloys. Figure 10(b_1_–b_3_) show the polarization curves of the alloys. It can be observed that the addition of zinc increases the width of the potential field zone (PFZ) and raises the corrosion potential between the PFZ and the matrix, thereby reducing corrosion resistance. This alloy exhibits the best corrosion resistance in the over-aged state and the worst corrosion resistance in the peak-aged state. Xu et al. [122] investigated Zn’s effect on Al-Mg-Si alloys and found that it enhanced the peak strain hardening effect. Tensile curves revealed a complex property evolution, in which strength and elongation initially increased together with Zn content. At 2.0 wt.% Zn, strength decreased significantly while elongation improved; conversely, at 6.0 wt.% Zn, strength surged sharply at the expense of elongation. Microstructural analysis for Zn contents of 0, 0.26, 0.6, 1.0, and 2.0 wt.% showed that β″ remained the dominant precipitate, with no η′ phase detected, indicating that initial property changes were governed by modifications within the β″ precipitation system. At 6.0 wt.% Zn, the precipitate structure shifts to the η′ phase, which exhibits good interfacial coherency with the matrix. In the 6.0Zn alloy, the microstructure contains a high density of β″ precipitates alongside a minor fraction of η′ precipitates, which is consistent with the results of Dang et al. [123]. Consequently, Zn addition enhances tensile strength and hardness via solid-solution strengthening, but its effect on toughness is more complex.

#### 4.2.3. The Effect of Other Alloying Elements on Al-Mg-Si Series Alloys

Despite its high electrical conductivity, the Al-0.13 wt.% Zr alloy wire exhibits a tensile strength of only 160–180 MPa [124], which does not meet the requirements for AAAC (All Aluminum Alloy Conductor) cables. Incorporating Zn into Al-Mg-Si alloys is expected to improve their mechanical strength and high-temperature performance, representing a key strategy for advancing alloy capabilities. Fu et al. [125] studied the effect of Zr addition on the properties of Al-Mg-Si alloys, and they found that the Al-Mg-Si-0.13Zr alloy exhibited good electrical conductivity, tensile strength, and heat resistance. Dang et al. [126] studied the effect of Zr on Al-Mg-Si-Mn alloys and found that 0.2% Zr refined the grain structure, promoting a transition from dendritic to equiaxed growth and concomitantly increasing elongation to 11.97%. Werinos et al. [126] reported that the addition of 100 ppm Sn can simultaneously achieve ultra-fast aging kinetics and high hardness peaks. Liu et al. [127] found that microalloying with Sn in the Al-0.103Mg-0.68Si alloy at 260 °C significantly refines the microstructure of the precipitated phase.

#### 4.2.4. The Effect of Rare Earth Elements on Al-Mg-Si Series Alloys

Recent studies have extensively explored rare earth elements for tailoring the microstructures and interfaces of aluminum alloys. Zhang et al. [128] found that Sc doping lowers the interfacial energy of the θ′/Al interface, while Lv et al. [129] computationally compared La, Ce, Nd, and Sc for strengthening the Mg_2_Si/Al interface. Calculations show that random doping with La, Ce, Nd, and Sc can promote the Al (100)/Mg_2_Si(100) interface and enhance its stability, making crack initiation at the interface more difficult and thereby enhancing the strength and toughness of 6000 series aluminum alloys.

Sc strengthens aluminum alloys mainly through the formation of the coherent L12-ordered Al_3_Sc phase [130]. This phase precipitates at higher temperatures and exhibits greater thermal stability (above 300 °C) than conventional β-Mg_2_Si nano-precipitates [131]. Furthermore, nano-dispersed L12 particles hinder dislocation movement, improving mechanical properties. Li et al. [132] found that Sc delays the overall precipitation kinetics by forming Sc-Cu clusters, thereby increasing thermal stability.

**Figure 11:**
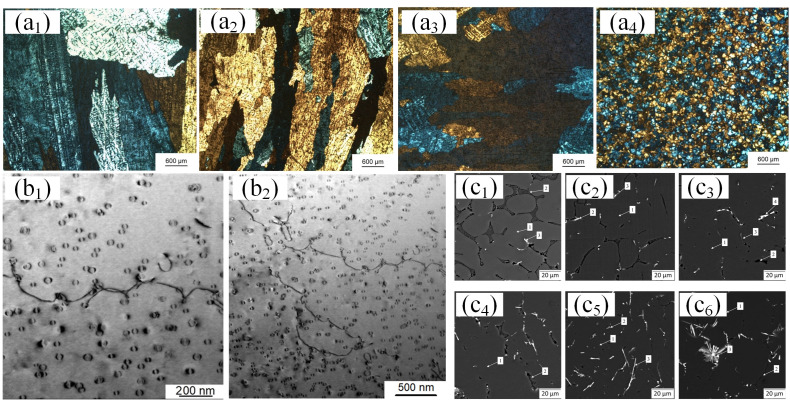
Grain structure after casting: (**a_1_**) Al0.3Mg1Si, (**a_2_**) Al0.3Mg1Si0.3Sc, (**a_3_**) Al0.3Mg1Si0.15Zr, and (**a_4_**) Al0.3Mg1Si0.3Sc0.15Zr. (**b_1_**,**b_2_**) (AlSi)3ScZr particles with the L12 structure [133]. Backscattered SEM micrographs showing as-cast microstructural evolution of Al—4 wt.% Mg—0.5 wt.% Si alloy with increasing in La content: (**c_1_**) 0 La; (**c_2_**) 0.1La; (**c_3_**) 0.25La; (**c_4_**) 0.50La; (**c_5_**) 0.75La; (**c_6_**) 1.0La [134].

**Figure 12:**
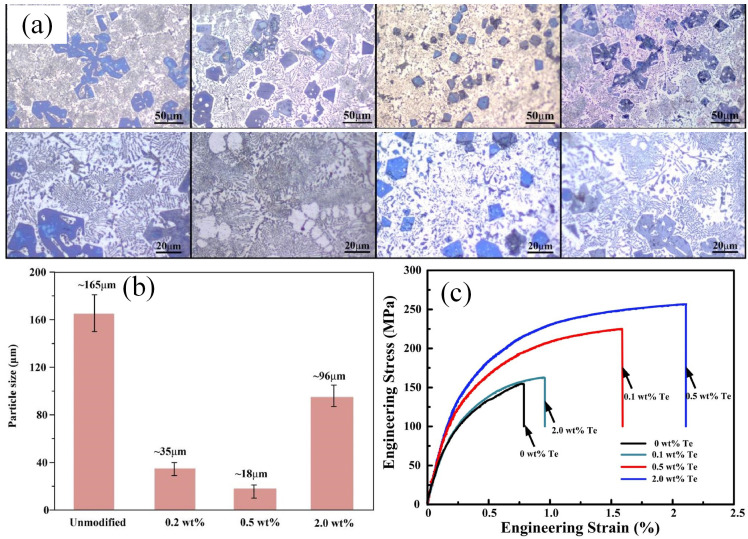
(**a**) The microstructure of the samples containing various contents of Te. (**b**) The average particle sizes of primary Mg_2_Si. (**c**) Engineering stress–strain curves plotting the tensile behavior of Al-20Mg-2Si alloys with varying Te contents [135].

In Al-Mg-Si alloys, the key strengthening phase Mg_2_Si typically solidifies as coarse dendrites during conventional casting, impairing mechanical performance. Achieving high-performance alloys thus requires precise control of Mg_2_Si’s morphology and size [133,134]. Modification offers a highly cost-effective method to simultaneously refine and tailor its structure. Evgenil et al. [133] investigated the effects of trace amounts of Sc (0.1–0.2 wt.%) and Zr (0.1–0.2 wt.%) on the as-cast microstructure of a silicon-rich Al–0.3Mg–1Si alloy; the combined addition of Sc and Zr significantly refined the grain size (Figure 11(a_1_–a_4_)). The combined addition of Sc and Zr formed (L12-type) Al_3_(Sc,Zr) dispersion phases within the grains and at the grain boundaries (Figure 11(b_1_,b_2_)), significantly refining the α-Al grain structure and verifying the grain-refining effect of Sc/Zr on the microstructure of Al–Mg–Si alloys. Vladislav et al. [134] investigated the effect of La addition on the microstructure of Al-Mg-Si alloys, as shown in Figure 11(c_1_–c_6_). Notably, the addition of 0.25 wt.% La almost completely eliminated the presence of the Mg_2_Si phase. The microstructure of the L2 alloy reveals that the AlLaSi phase is located in the vicinity of very fine eutectic Mg_2_Si phases. This partially confirms the “absorption effect” of the La-rich phase. This effect may have suppressed the anisotropic growth of the Mg_2_Si phase, thereby altering its shape factor. The addition of 0.1 wt.% La resulted in finer dendrites of the Mg_2_Si phase, which nevertheless retained a lamellar morphology. Li et al. [135] modified Mg_2_Si in the Al-20Mg-2Si alloy by adding Te elements, and they observed a non-monotonic influence of Te on the primary Mg_2_Si phase. As Te increases from 0 to 0.6 wt.% [Figure 12a], Mg_2_Si evolves from coarse dendrites into refined polygonal/funnel-shaped crystals (~18 μm). However, at 2.0 wt.% Te, the structure reverts to dendritic (~106 μm), nearly losing all modification. This refinement correlates with increased tensile strength, rising from 162 MPa to 267 MPa at 0.6 wt.% Te. The modification mechanism of Te involves both promoting the heterogeneous nucleation of primary Mg_2_Si and acting as a doping agent within the growing phase. The introduction of Te promotes the formation of the Al_2_Te_3_ phase, which serves as the heterogeneous nucleus of the primary phase. The modification effect achieved with 2.0 wt.% Te ultimately fails because the Te-containing compounds precipitate prior to the Mg_2_Si phase, depleting available Te and hindering effective modification.

Aluminum alloys have a very high recycling rate, with Fe being the primary impurity concern in recycled Al-Si alloys. Fe impurities form Fe-rich intermetallic compounds such as θ-Al_13_Fe_6_, α-Al_8_Fe_2_Si, β-Al_6_FeSi, δ-Al_4_FeSi_2_ and π-Al_8_FeMg_3_Si_2_, which often tend to form needle-like or plate-like structures at high temperatures [136]. Since the iron-rich phases cannot be altered by subsequent heat treatment, considerable research has focused on controlling their formation from the melt. Additionally, Fe in die-cast components not only necessitates the use of pure Al for preparation but also accelerates mold wear. Therefore, developing high-strength, high-toughness aluminum alloys that tolerate higher Fe levels is crucial for reducing production losses and significantly promoting the recycling of aluminum scrap. Recently, Ce has attracted significant research interest as an alloying addition for aluminum alloys. Studies indicate that even small amounts of Ce can impart beneficial effects in secondary Al-Si-Mg-(Fe) alloys. For example, Michael et al. [137] demonstrated that 0.26–0.6% Ce enhances the ductility of high-iron recycled Al-Si-Mg alloys by 60%, with elongations exceeding 6%. The best performance, including 6.2% EL and 236 MPa UTS, was obtained with 0.26% Ce, attributed to the suppression of the π-Al phase. Peng et al. [138] studied the mechanism by which Ce influences Al-Mg-Si aluminum alloys, and they found that Ce-containing alloys exhibit superior tensile properties after extrusion. Ce promotes the transformation of heavily deformed grain substructures during hot extrusion, reducing the fraction of large-deformation grains and increasing the proportion of substructures in the alloy. After aging at 170 °C for 12 h, the Ce-modified alloy achieved a UTS of 602 MPa, a YS of 336 MPa, and an EL of approximately 16% compared to 371 MPa, 326 MPa, and 16.7% for the Ce-free alloy. The enhancement in tensile and yield strength with Ce addition is primarily attributed to its role in promoting age-hardening precipitation within the alloy, resulting in more and finer precipitates. Fracture surface analysis reveals that the Ce-free alloy exhibits more intergranular cracking and larger, deeper dimples than the Ce-doped alloy, which may explain its slightly higher plasticity. In summary, Ce enhances alloy performance by promoting beneficial phase precipitation.

In automotive and aerospace applications, Al-Mg-Si alloys are valued for their superior formability and conductivity, but their service life is often limited by corrosion. High Si/Mg ratios are detrimental to corrosion resistance because excess Si segregates to grain boundaries, thus initiating intergranular corrosion [139]. Microalloying with rare earth elements has emerged as a promising strategy to enhance corrosion resistance. Zheng et al. [140] studied the influence of La on corrosion behavior and found that La improves corrosion resistance. After immersion in 3.6% NaCl solution, the La-added alloy showed clearly markedly less severe attack than the alloy without La. Notably, after 26 h immersion, Mg_2_Si-phase particles were almost absent from corrosion pits in the La-modified alloy, indicating that La suppresses the dissolution of Mg_2_Si. Additionally, a La-rich shell on Mg_2_Si particles can limit the dissolution of Mg within the particles, thereby mitigating corrosion of the surrounding aluminum matrix. Yuan et al. [141] found that La improves the conductivity and heat resistance of AA6201 aluminum alloys. Wang et al. [142] employed multi-alloying with Ce, Sc, and Y to enhance the heat and corrosion resistance of Al-Mg-Si alloys. The research identified specific contributions: Sc improved heat resistance, Ce enhanced corrosion resistance, and a synergistic Sc + Y co-addition optimized both properties simultaneously.

In conclusion, rare earth elements significantly modify Al-Mg-Si alloys, with each element imparting a unique influence through a different mechanism. Scandium contributes potent strengthening through coherent Al_3_Sc precipitates. Tellurium modifies the morphology of the primary Mg_2_Si phase. Cerium notably improves the ductility of iron-containing recycled cast alloys. La increases the matrix fraction of Mg_2_Si, thereby enhancing corrosion resistance. This spectrum of effects allows for precise alloy design.

## 5. The Effect of Nanoparticles on the Microstructure and Properties of Aluminum Alloys

Although alloying improves the performance of aluminum alloys, it has certain limitations. For example, excessive addition of alloying elements can lead to segregation, and adding multiple elements in excessive quantities can complicate precipitation phases and sequences, ultimately affecting an alloy’s performance. In contrast, nanoparticles represent a new type of strengthening agent that offers high thermal stability, pronounced strengthening effects, and favorable cost efficiency. Nanoparticles play a key role in aluminum alloys by refining the microstructure, enhancing mechanical strength, and improving fatigue resistance and other service properties. These combined advantages make nanoparticle-reinforced alloys particularly suitable for critical applications in aerospace and new energy vehicles.

### 5.1. Types of Nanoparticles and Preparation Methods

Metal matrix composites may be reinforced by various types of nanoparticles, and their performance can be significantly improved by reasonably controlling the amounts of nanoparticles. Common types of nanoparticles include carbides (TiC [143], SiC [144], and B_4_C [145]), oxides (Al_2_O_3_), nitrides (TiN and AlN) and borides (TiB_2_). Table 4 shows the physical properties of some particles. For example, TiC particles are used to reinforce aluminum-based composite materials to enhance their mechanical properties and wear resistance. Al_2_O_3_ [146] is commonly used to improve the thermal stability and wear resistance of aluminum alloys due to its high-temperature stability and hardness. TiN [147] and AlN [148] particles can improve the high-temperature performance and wear resistance of aluminum alloys. TiB_2_ [149] nanoparticles can be added to aluminum alloys in different ways. Common ceramic particles in aluminum alloys exhibit distinct physical properties (Table 1), leading to various performance characteristics in alloys. The incorporation of various nanoparticles imparts distinct modifications to aluminum alloys, notably affecting their strength, hardness, tribological behavior, castability, and corrosion resistance. By optimizing distribution, size, and content, the performance of aluminum alloys can be further enhanced.

The introduction of nanosized reinforcing particles can enhance the performance of an aluminum alloy matrix. Compared with traditional aluminum alloys, nanoparticle-reinforced aluminum alloys exhibit superior mechanical properties and hold great promise for applications in new energy vehicles, aerospace, and other fields. However, achieving uniform dispersion of nanoparticles within the matrix remains a significant technical challenge. Currently, based on the different origins and characteristics of the particles, the preparation techniques for nanoparticle-reinforced aluminum alloys can be broadly categorized into two categories, exogenous addition and endogenous synthesis. A combined approach that integrates the advantages of both methods involves first preparing a medium using the in situ synthesis method, followed by dispersion using the direct addition method.

Exogenous addition includes processes such as stir casting [152], powder metallurgy [153], and additive manufacturing [154]. The advantage of the direct addition method is that the preparation of ceramic particles is not affected by the matrix alloy, allowing for flexible adjustment of the size, morphology, and volume fraction of the nanoparticles. In the external addition method, improving the dispersion of nanoparticles, enhancing their interface bonding with the matrix, and avoiding contamination are key issues. To address these issues, researchers have employed technologies, such as solvent-assisted dispersion combined with mechanical ball milling, to achieve uniform dispersion of nanoparticles and optimized ball milling process parameters to break down oxide film on particle surfaces, thereby improving interfacial bonding. Stir casting introduces reinforcing particles to continuously stirred molten metal to achieve uniform dispersion. The equipment required for this method required is simple and relatively low-cost, but high-temperature exposure can cause interfacial reactions that weaken bonding strength. Powder metallurgy is a widely used technique for preparing nanoparticle-reinforced aluminum alloys. It offers advantages such as flexible selection of reinforcing phases, precise control of content, and strong design flexibility. By adjusting process parameters, the performance of composite materials can be optimized to meet specific application requirements. Additive manufacturing technology (3D) designs a three-dimensional part structure using computer-aided design and then builds the component layer-by-layer using a laser or electron beam to melt and solidify the composite material [155]. It has been the fastest-growing, most flexible, and most widely applicable method for preparing aluminum–matrix composite materials in the past decade. Compared with traditional manufacturing technologies, it enables the fabrication of complex structures (e.g., lattices and internal voids) and finds widespread application in aerospace, medical, automotive, and other fields.

Compared with the ex situ method, the in situ method offers significant advantages for preparing nanoparticle-reinforced aluminum alloys. The in situ method directly generates nanoparticles within the aluminum alloy matrix, resulting in stable thermochemical properties, resistance to decomposition, and the absence of brittle intermediate phases. This method effectively prevents interfacial contamination and yields a uniform particle distribution with excellent interfacial bonding. Typical in situ preparation methods include combustion synthesis, direct melt reaction, and molten salt-assisted methods.

The intermediate alloy method is a widely used technique for the preparation of nanoparticle-reinforced metal matrix composites, particularly aluminum alloys. This approach combines the advantages of both the ex situ and in situ methods, involving pre-preparing an intermediate alloy containing nanoparticles and then adding the intermediate alloy to the matrix alloy. During melting and solidification, the nanoparticles are uniformly dispersed within the matrix alloy. The process is relatively simple and suitable for industrial-scale production. A key advantage is its ability to ensure a homogeneous nanoparticle distribution by controlling particle size, thereby successfully mitigating agglomeration and settling. By adjusting the amount of the intermediate alloy added, a high volume fraction of nanoparticles can be achieved. This method holds broad application prospects in the field of nanoparticle-reinforced aluminum alloys.

### 5.2. Control of the Microstructure of Aluminum Alloys by Nanoparticles

The regulation of the microstructure of aluminum alloys by nanoparticles represents a profound transformation ranging from the atomic scale to macroscopic properties. Its core lies in actively intervening and precisely controlling the microstructural evolution during alloy solidification, solid-state phase transformations, and hot working by introducing exogenous or in situ nanoscale second phases. This achieves comprehensive optimization of grain size, precipitation phase distribution, and eutectic morphology, leading to enhanced overall performance. Far from a simple physical addition, this process constitutes a complex system engineering challenge that involves thermodynamics, kinetics, and interfacial science. First, the most significant effect of nanoparticles lies in the extreme refinement of primary α-Al grains. At the onset of solidification, nanoparticles such as TiB_2_, TiAl_3_ or TiC serve as highly effective heterogeneous nucleation substrates due to their excellent lattice matching with α-Al. Dispersed uniformly in the melt, they drastically lower the energy barrier for α-Al nucleation, increasing the nucleation rate by orders of magnitude. Consequently, traditional coarse columnar or dendritic structures are replaced by a uniform, fine-grained equiaxed microstructure. This follows the classical heterogeneous nucleation theory, with nanoparticles, due to their extremely high specific surface area and number density, pushing the theory to its practical limits. Moreover, nanoparticles act as “pinning agents” during grain growth. According to the Zener pinning model, the drag force that particles exert on grain boundaries is proportional to their volume fraction and inversely proportional to their radius. Thermally stable nanoparticles thus effectively pin migrating grain boundaries, strongly suppressing abnormal grain growth and maintaining microstructural stability during subsequent heat treatment or hot working processes.

#### 5.2.1. The Effect of Nanoparticles on the Solidification Behavior of Aluminum Alloys

**Figure 13:**
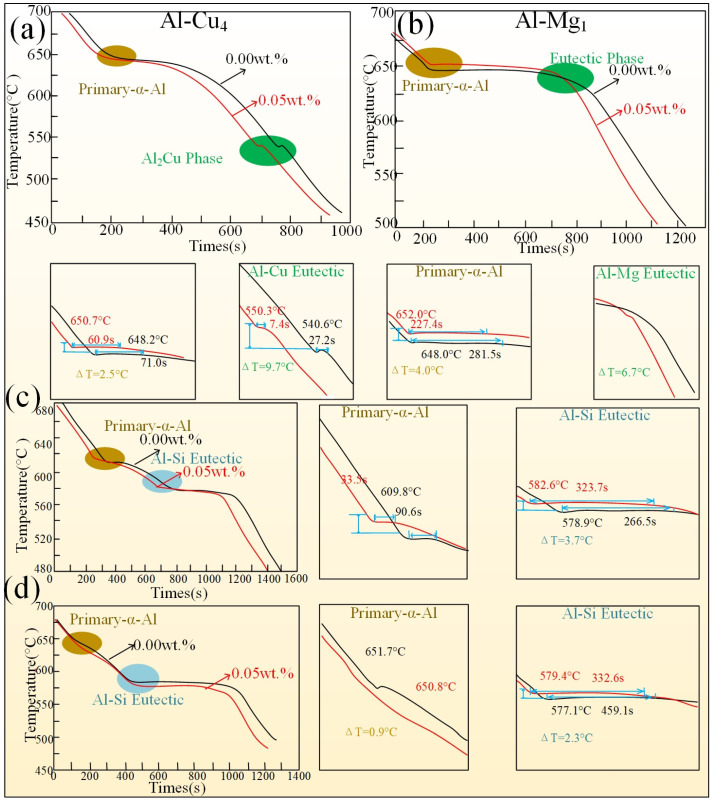
(**a**) Cooling curves of Al-4Cu alloys. (**b**) Cooling curves of Al-1Mg alloys [159]. (**c**) Cooling curves of Al-7Si alloys. (**d**) Cooling curves of Al-17Si alloys [160].

In recent years, extensive research has demonstrated that the introduction of nanoparticles (such as TiC, TiB_2_, and other nanoceramics) is an effective strategy for achieving ultra-fine grain refinement and microstructural homogenization. The underlying mechanism involves reconfiguring solidification kinetics through the regulation of interfacial behavior and solute fields during nucleation and growth stages. Taking Al-based composites with in situ formed nanoparticles as an example, studies show that high-density nanoparticles significantly reduce the undercooling required for α-Al nucleation, promote multi-site heterogeneous nucleation, and transform the as-cast microstructure from coarse columnar grains to fine equiaxed grains, thereby achieving highly efficient grain refinement [156]. Research on aluminum alloys modified with trace amounts of nanoparticles indicates that during solidification, some nano-TiB_2_ particles adsorb at the solid–liquid interface, impeding heat and solute exchange, inhibiting crystal orientation growth, significantly reducing grain size, and promoting a more uniform microstructure. Concurrently, they enhance alloy strength through thermal mismatch and dislocation strengthening [157]. Further modeling of traditional Al–Ti–B/Al–Ti–C particle systems reveals that the dissolution–reprecipitation and “push/swallow” behavior of nanoscale TiB_2_ particles in the melt decisively influence the particle size distribution and grain refinement effect, ultimately determining their effectiveness as nucleation sites. On the other hand, studies utilizing stirred casting or powder metallurgy to uniformly disperse TiC nanoparticles indicate that thoroughly breaking agglomerates and enhancing nanoparticle dispersion in the melt are crucial for realizing their grain refinement potential. When TiC nanoparticles are uniformly distributed, the spacing between α-Al secondary dendrite arms significantly decreases, and solidification defects such as shrinkage cavities and hot cracks are also reduced [158]. Under extreme cooling conditions, such as in additive manufacturing, nanoprecipitates/-particles like Al_3_Ta formed in nano-functionalized aluminum alloys have been further demonstrated to provide stable nucleation sites under high temperature gradients and rapid cooling rates, inhibiting the formation of columnar crystals and texture, resulting in fine equiaxed microstructures and isotropic mechanical properties. Researchers have also modified the solidification behavior of aluminum alloys by incorporating nanoparticles. For instance, Yao et al. [159] demonstrated that adding nanocrystals to Al-Cu_4_/Al-Mg_1_ alloys elevated nucleation temperatures and reduced crystal growth times (Figure 13a,b). Furthermore, it also elevated the nucleation temperature of the eutectic phase. Song et al. [160] studied the effects of Fe-B-Si nanocrystals on Al7Si and Al17Si alloys and found that nanocrystal incorporation increased the nucleation temperatures of both primary Si and eutectic Si (Figure 13c,d). Ma et al. [161] investigated the effect of TiC-TiB_2_ nanoparticles on the solidification curves of Al-Cu alloys and found that nanoparticle addition increased nucleation temperature and accelerated cooling rates, as shown in Figure 3 of the paper by Ma et al. As an effective grain refiner, nanoparticles can suppress the coarsening of α-Al dendrites during crystallization. Chen et al. [162] investigated the effect of TiC particles on the solidification behavior of AA6063 alloys. The results indicate that by facilitating gradual latent heat release and enhancing melt flowability, particle manipulation thereby reduces thermal cracking in castings. Based on these findings, the prevailing view is that nanoparticles achieve synergistic control over grain size, morphology, and as-cast defects in aluminum alloys at the solidification scale. This is accomplished by modifying the heterogenous nucleation barrier of α-Al, regulating the “push–pull” behavior between particles and solid–liquid interfaces, modulating local thermal/solute fields, and inhibiting dendrite growth. This approach provides a novel microstructural design pathway for the casting and additive manufacturing of high-performance aluminum alloys.

#### 5.2.2. The Effect of Nanoparticles on the Eutectic Phase of Aluminum Alloys

**Figure 14:**
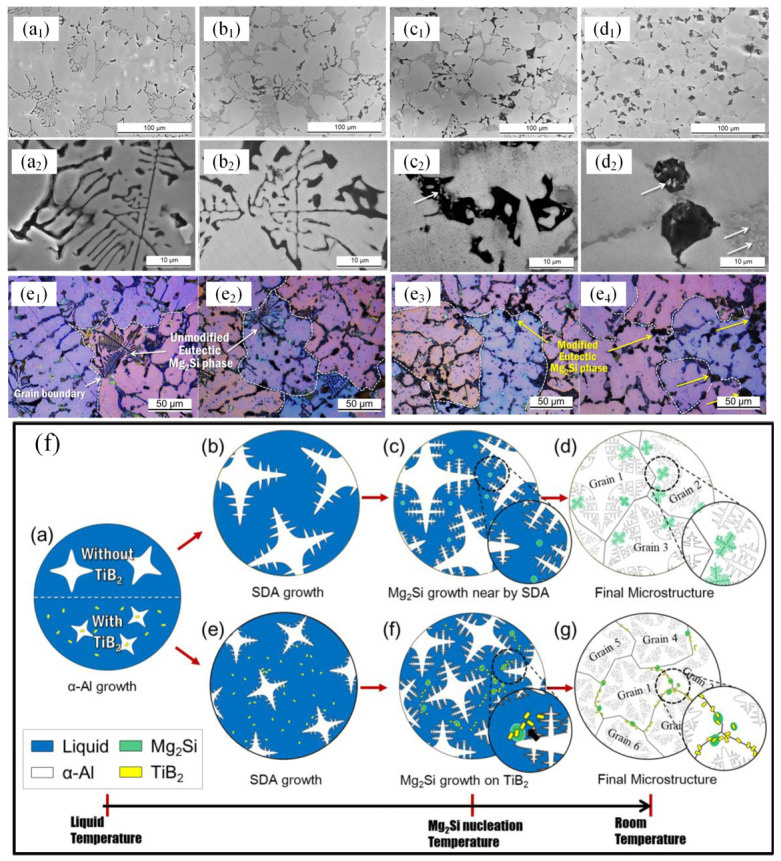
SEM images of the eutectic Al + Mg2Si phases of the Al–8Zn–6Si–4Mg–2Cu alloy with different Ti amounts: (**a_1_**,**a_2_**) without Ti; (**b_1_**,**b_2_**) at 0.1% of Ti; (**c_1_**,**c_2_**) at 0.5% of Ti; and (**d_1_**,**d_2_**) at 1% of Ti. Polarized microscope images (high magnification) of the Al–8Zn–6Si–4Mg–2Cu alloys with different amounts of Ti: (**e_1_**) without Ti; (**e_2_**) at 0.1% of Ti; (**e_3_**) at 0.5% of Ti; and (**e_4_**) at 1% of Ti. (**f**) Schematic presentation of the solidification processes of eutectic Al + Mg_2_Si phases in aluminum alloys: (a) → (b) → (c) → (d) without TiB_2_ particles; (a) → (e) → (f) → (g) with TiB_2_ particles [167].

In widely used cast aluminum alloys such as A356 and A380, the eutectic reaction (liquid phase → α-Al + silicon phase) constitutes the final stage of solidification. In untreated alloys, eutectic silicon tends to precipitate in coarse flake/needle-like or character-like forms. This morphology originates from the strong anisotropic growth behavior of silicon crystals. These coarse, brittle silicon phases act as natural stress concentrators and crack-propagation pathways, severely fracturing the ductile aluminum matrix, leading to low elongation and poor impact toughness, and ultimately limiting the alloy’s application in high-performance structural components. To ensure favorable mechanical properties, eutectic Si must be modified into a fine fibrous morphology. The traditional modification relies on adding certain alloying elements [163,164], which must be carefully balanced in content and impact on other properties. In contrast, the incorporation of nanoparticles not only refines eutectic Si but also enhances multiple properties simultaneously [165,166]. Research indicates that nanoparticles effectively regulate eutectic Si growth. Kim et al. [167] investigated the effect of TiB_2_ nanoparticles on the Mg_2_Si eutectic phase. The results showed that the addition of TiB_2_ effectively improved the eutectic Mg_2_Si phase, causing changes in the particle size and morphology of the Mg_2_Si phase (Figure 14(a_1_–e_4_)). The morphology of the eutectic structure changed from coarse, irregular shapes to fine polygonal shapes. Figure 14f illustrates the mechanism by which TiB_2_ nanoparticles improve the morphology of Mg_2_Si. Jiang et al. [168] incorporated varying concentrations of SiC nanoparticles into Al-Si alloys and found that SiC significantly alters the microstructure of eutectic Si. As the SiC content increases, the refinement of eutectic Si becomes increasingly pronounced. SEM analysis reveals a clear morphological evolution from needle-like to short rod-like and even near-spherical shapes. Wang et al. [169] investigated the effect of TiC nanoparticles on eutectic Si in hypoeutectic Al-Si alloys. They found that nanoparticles refined the size of eutectic Si. Song et al. [170] added Fe-B-Si nanocrystals to Al-7Si and Al-17Si alloys. In the Al-7Si alloy, the addition of 0.05 wt.% Fe-B-Si reduced the size of eutectic Si and significantly improved its morphology. In the Al-17Si alloy, the addition of nanocrystals refined the eutectic Si and enhanced its size uniformity. Jiang et al. [171] reported that in situ synthesized TiC particles modified the eutectic Si in iron-rich eutectic Al-Si alloys. With increasing TiC particle content, the eutectic Si length significantly decreased, exhibiting the shortest length at 1 wt.% TiC. In hypereutectic alloys, nanoparticles refine both eutectic Si and primary Si. Cui et al. [172] introduced Al_2_O_3_ nanoparticles into the Al-20Si alloy, reducing the primary Si size by 80% and transforming eutectic Si from coarse flakes into fine coral-like particles. In summary, nanoparticles influence eutectic reactions and eutectic phases through two pathways, including direct intervention in nucleation and growth and indirect restructuring of macroscopic solidification patterns. By acting on both thermodynamic (reducing nucleation energy barriers) and kinetic (altering interfacial growth and segmenting the melt) aspects, nanoparticles transform harmful coarse brittle phases into beneficial fine ductile phases. This represents not merely a microstructural refinement but a fundamental redesign of material properties involving phase transformation mechanisms. It enables cast aluminum alloys to overcome the strength–ductility trade-off, providing a robust technological foundation for meeting the stringent demands for lightweight construction and high safety in new energy vehicles. Moving forward, developing multifunctional composite nanoparticle systems to achieve smarter, more controllable customization of eutectic phase morphology, size, and distribution will be a key direction for advancement in this field.

#### 5.2.3. The Effect of Nanoparticles on Precipitation Phases in Aluminum Alloys

Nanoparticles have transformed the size, distribution, and stability of strengthening precipitates in aluminum alloys. In heat-treatable aluminum alloys, strength primarily originates from intermetallic compounds (e. g, Al_2_Cu, Mg_2_Si, and Al_2_CuMg) that precipitate during aging. In traditional processes, precipitates tend to nucleate unevenly at defects such as dislocations and grain boundaries, leading to coarsening and agglomeration that degrade performance. However, introduced nanoparticles, particularly those that are coherent or partially coherent with the matrix and remain stable at the solid-solution temperature of aluminum alloys, such as Al_3_(Sc, Zr) phases containing Sc and Zr, provide a large number of uniformly distributed heterogeneous nucleation sites for strengthening precipitates. Current research generally indicates that introducing nanoparticles into aluminum alloys can significantly regulate precipitation behavior during aging, achieving synergistic effects between precipitation strengthening and nanoparticle strengthening. Pu et al. [173] incorporated 1 vol% SiC nanoparticles into a 7075 matrix. After T6 treatment, the η′ phase (MgZn_2_) exhibited a smaller size and more uniform distribution, while the dislocation density increased. Li et al. [174] systematically investigated the effects of different SiC nanoparticle contents on Al-Zn-Mg-Cu alloys. The results indicate that SiC refines and increases the number density of η′/η precipitates, significantly enhancing peak aging strength, consistent with the research by Pu et al. Graphene nanoparticles also effectively refine the precipitates. Li et al. [175] fabricated 2024Al-GNP composites by employed spark plasma sintering. The uniformly distributed graphene nanosheets provided heterogeneous nucleation interfaces while introducing abundant dislocations, significantly promoting the precipitation and refinement of θ(Al_2_Cu) phases. Simultaneously, phase-field simulations further characterized the precipitation evolution process. Zhou et al. [176] incorporated TiC particles into the melt. Microscopic observations revealed that nano-TiC both refined α-Al grains and increased the precipitation amount while reducing the size of θ′ (Al_2_Cu) phases, significantly enhancing yield strength and ductility. Ma et al. [177] added TiC-TiB_2_ nanoparticles to Al-Cu alloys. After T6 treatment, compared to the untreated alloy, the nanoparticle-modified alloy showed smaller precipitate sizes, higher precipitate numbers, and more uniform distribution. Tian et al. [178] discovered that nano-TiC induced the formation of abundant fine θ′ precipitates with a more uniform distribution, which is crucial for achieving high strength and toughness. These precipitates and nanoparticles form a composite strengthening network at grain boundaries and within the matrix. Tian et al. [179] further compared the effects of single-size versus dual-phase (micron + nanometer) TiC_p_ particles on creep behavior and θ′ precipitation in Al–Cu alloys. Dual-phase TiC_p_ particles induced a higher density and finer θ′ precipitates, enhancing creep resistance by 10 to 38 times. This demonstrates that particle size and distribution can precisely regulate precipitation strengthening effects at elevated temperatures. Liu et al. [180] demonstrated that TiB_2_ particles increased the number density of η′/η phases, enabling higher yield strength and improved plasticity when combined with appropriate heat treatment. Zeng et al. [181] incorporated in situ nano-TiC particles into 2219 aluminum alloys. The TiC particles enhanced precipitation hardening, increasing the volume fraction and reducing the size of the second phase during peak aging, significantly improving the tensile properties of the deposited layer. A common mechanism emerges from the above studies, in which nanoparticles reconfigure the formation and evolution of strengthening phases in aluminum alloys by providing heterogeneous nucleation sites, inducing dislocations and lattice distortions, inhibiting grain growth, and stabilizing interfaces. This provides new insights for the design of high-strength, high-toughness, and high-temperature-service aluminum alloys.

### 5.3. The Effect of Nanoparticles on the Properties of Al-Si-Mg Alloys

**Figure 15:**
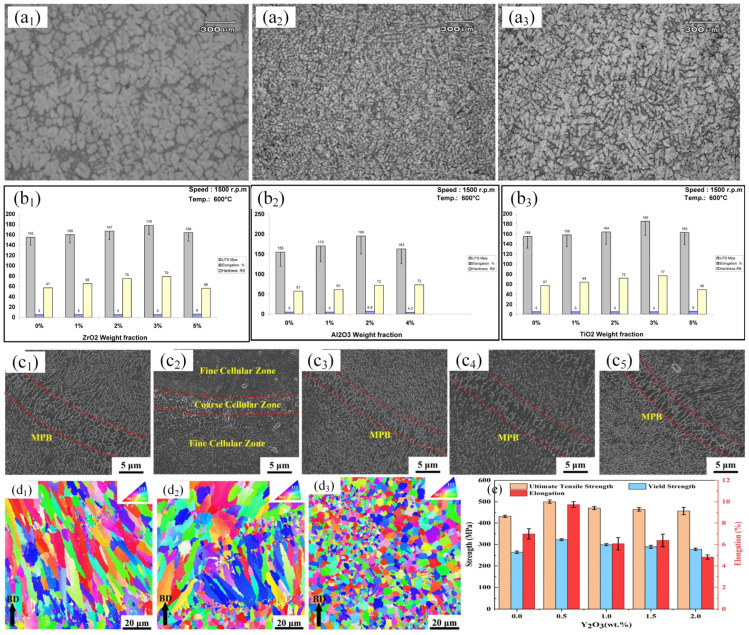
(**a_1_**) Microstructure of the A356 matrix alloy. (**a_2_**) Microstructure of A356 with Al_2_O_3_. (**a_3_**) Microstructure of A356 with TiO_2_. (**b_1_**) The effect of ZrO_2_ nanoparticles% on the UTS, hardness and ductility. (**b_2_**) The effect of Al_2_O_3_ nanoparticles% on the UTS, hardness and ductility. (**b_3_**) The effect of TiO_2_ nanoparticles% on the UTS, hardness and ductility [190]. SEM images of SLMed samples: (**c_1_**) 0% Y_2_O_3_; (c_2_) 0.5% Y_2_O_3_; (**c_3_**) 1.0% Y_2_O_3_; (c_4_) 1.5% Y_2_O_3_; (**c_5_**) 2.0% Y_2_O_3_. EBSD orientation maps of the Al grains across their building direction in (**d_1_**) 0% Y_2_O_3_, (**d_2_**) 2.0% Y_2_O_3_, and (**d_3_**) 0.5% Y_2_O_3_. (**e**) Ultimate tensile strength, yield strength, and elongation data of the SLMed AlSi10Mg and Y_2_O_3_-AlSi10Mg alloys [191].

Nanomaterials serve as excellent reinforcing agents for aluminum alloys. Studies indicate that ceramic particles can serve as effective modifiers, refining grain structure or altering silicon-phase morphology while avoiding detrimental interfacial reactions [182,183]. Among reinforcement particles, TiB_2_ particles excel at simultaneous grain refinement and Si-phase modification, though coupling agents improve the distribution. TiC particles, due to their structural similarity to α-Al and strong metallic bonding, achieve uniform dispersion and excellent interfacial adhesion. Such particulate additions fundamentally alter the solidification behavior of the alloy [184,185,186]. After adding particles, the onset temperatures of α-Al nucleation and various eutectic reactions increase while the nucleation undercooling decreases. Particles promoted heterogeneous nucleation and increased nucleation efficiency. However, only about 1% of particles typically serve as effective heterogeneous nucleation sites [187]. According to adsorption model theory, particles that cannot serve as nucleation sites are adsorbed onto the solid–liquid interface and adhere to the growing α-Al front [188,189]. Particles with low thermal conductivity act as effective thermal barriers, impeding the dissipation of latent heat during solidification and thereby suppressing the growth of α-Al dendrites. Iman S et al. [190] investigated the effects of Al_2_O_3_, TiO_2_, and ZrO_2_ nanoparticles on the microstructure and properties of the A356 aluminum alloy. As shown in Figure 15(a_1_–a_3_), which illustrates the effects of Al_2_O_3_ and TiO_2_ nanoparticles on the microstructure of the A356 aluminum alloy, it can be seen that the addition of nanoparticles significantly refines the grain size of the alloy. Figure 15(b_1_–b_3_) show the effects of different nanoparticles on the properties of the A356 aluminum alloy. It was found that the addition of an appropriate amount of nanoparticles can enhance the properties of the aluminum alloy. Zhang et al. [191] investigated the effects of Y_2_O_3_ nanoparticles on the microstructure and properties of AlSi10Mg alloys produced by selective laser melting. Figure 15(c_1_–c_5_) show SEM images of the SLM specimens. Compared to the unmodified SLM specimens, the grains in the Y_2_O_3_-modified specimens are relatively finer (especially those at the center of the melt pool). The EBSD patterns in Figure 15(d_1_–d_3_) also illustrate this point. Figure 15e summarizes the mechanical properties, demonstrating that the addition of an appropriate amount of Y_2_O_3_ nanoparticles can significantly enhance the properties. Yao et al. [159] found that the grain size in Al-Cu_6_ and Al-Mg_1_ alloys refined with nanocrystalline particles decreased from 2016.8 micrometers and 1127 micrometers to 263.8 micrometers and 238.8 micrometers, respectively. Xi et al. [192] reported that incorporating submicron TiC particles into Al-Si-Mg alloys effectively increases YS and UTS. Enhanced mechanical properties are linked to grain refinement and modified Si morphology. The base alloy contains short-plate Si, which acts as a source of stress concentration. Adding 0.1 wt.% TiC reduces the average Si length and increases spheroidization, with a near-spherical morphology achieved at 0.3 wt.% TiC. The spheroidization of the Si phase alleviates stress concentration, thereby enhancing ductility. However, excessive TiC can form detrimental needle-like Al_3_TiSiₓCₓ phases, reducing ductility [193]. Therefore, an optimal TiCp content improves UTS, while the combination of spheroidization of the Si phase and grain refinement improves ductility.

Li et al. [194] studied C-TiB_2_ in Al-Si-Mg-Mn alloys and found that 0.6 wt.% addition significantly reduced the grain size to 210.7 μm, corresponding to increases of 13.8% in YS and 18.7% in EL. For Al-Si-Mg alloys, the precipitation of nanoscale age-hardening phases GP and β” during aging significantly improves mechanical properties. C-TiB_2_ nanoparticles further refine the size of these β” precipitates.

While TiC particles are thermodynamically unstable in Al-Si melts and can form harmful intermetallics, TiCN particles exhibit enhanced refining potency in Al-Si-Mg alloys [195]. TiB_2_ particles possess higher thermodynamic stability than TiC. Combining the two, two-phase nanoparticles more effectively regulate Al-Si-Mg alloys. Li et al. [196] demonstrated potent grain refinement in Al-10Si-2Mg alloys using in situ TiC-TiB_2_ particles: 0.1% addition reduced the grain size from 890 to 468 μm (67.6% reduction), and 0.3% addition achieved 201 μm (77.6% reduction). Moreover, the composite containing TiC-TiB_2_ particles exhibited simultaneous improvements in both strength and ductility compared to the unreinforced material. Additionally, the alloy reinforced with these two-phase nanoparticles exhibited superior strength and ductility after LTST treatment.

**Figure 16:**
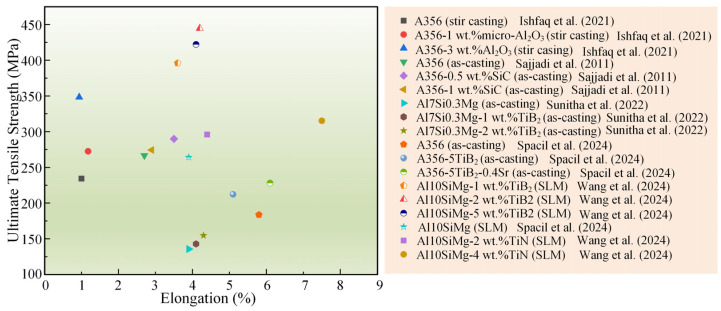
Performance of nanoparticle-enhanced aluminum alloys [198,199,200,201,202].

Fatigue resistance is a critical property of structural alloys. Liu et al. [197] utilized in situ nanocrystals to optimize the fatigue performance of Al-7Si-0.3Mg composite materials. They observed significantly refined α-Al, eutectic Si, Mg_2_Si, and β” phase. Most importantly, the in situ nanocrystal-optimized composite exhibited a sixfold increase in fatigue life compared to the unoptimized material, both at 120 MPa (60 Hz) and at 260 MPa (20 Hz). In summary, nanoparticles exert multifaceted impacts on aluminum alloys. They promote grain refinement and alter the size and distribution of precipitates, improving the strength and hardness of aluminum alloys. Notably, even slight nanoparticle addition can achieve better performance. Figure 16 summarizes the key properties of selected nanoparticle-reinforced aluminum alloys.

### 5.4. The Effect of Nanoparticles on the Properties of Al-Mg-Si Alloys

**Figure 17:**
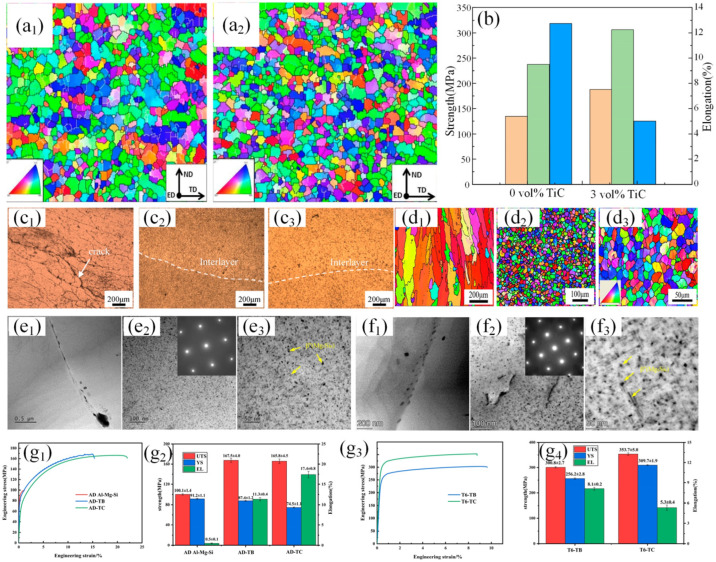
EBSD images of (**a_1_**) AA6005A-HEBM and (**a_2_**) AA6005A-HEBM-3 vol% TiC. (**b**) Tensile testing results of the AA6005A-HEBM and AA6005A-HEBM-3 vol% TiC [205]. OM microstructure: (**c_1_**) Al-Mg-Si, (**c_2_**) Al-Mg-Si-TiB2, and (**c_3_**) Al-Mg-Si-TiC. EBSD images and corresponding grain size distributions of three alloys: (**d_1_**) Al-Mg-Si alloy, (**d_2_**) Al-Mg-Si-TiB2; (**d_3_**) Al-Mg-Si-TiC. TEM images of the T6- Al-Mg-Si-TiB2. (**e_1_**) BF image showing the precipitates on the grain boundary and in the grain. (**e_2_**,**e_3_**) BF image and corresponding SAED pattern showing the precipitates in the grain. TEM images of the T6- Al-Mg-Si-TiC: (**f_1_**) BF image showing the precipitates on the grain boundary and in the grain; (**f_2_**,**f_3_**) BF image and corresponding SAED pattern showing the precipitates in the grain. Stress–strain curves and the mechanical property results of three as-deposited alloys: (**g_1_**) stress–strain curves; (**g_2_**) mechanical property results. Stress–strain curves and the mechanical property results of two T6-treated alloys: (**g_3_**) stress–strain curves; (**g_4_**) mechanical property results [207].

Extensive research on nanoparticle-modified Al-Mg-Si alloys in recent years is advancing the development of high-performance materials, which is crucial for energy-efficient vehicle technologies. Wang et al. [203] found that in TRC Al-Mg-Si alloys, bands with a lower Mg/Si ratio exhibited severe centerline segregation, whereas those with higher ratios showed no obvious segregation band. TiC particles significantly enhance the nucleation efficiency of α-Al, generating numerous fine grains [204] and effectively mitigating center segregation. The formation of fine equiaxed grains increases boundary density to limit solute aggregation, while nanoparticles predominantly segregate at the solid–liquid interface to form a diffusion-blocking layer, collectively restricting the segregation of Mg and Si [205]. Ferijoo et al. [206] investigated the effects of TiC nanoparticles on the microstructure and mechanical properties of extruded 6005A aluminum alloy sheets. The study showed that the addition of TiC nanoparticles significantly refined the grain size (Figure 17(a_1_,a_2_)), reducing it from 745 nm to 609 nm. In terms of mechanical properties, it significantly improved the yield strength and tensile strength, but reduced ductility (Figure 17b). Li et al. [207] (“Microstructure and Mechanical Properties of TiB_2_/TiC Particle-Modified Al-Mg-Si Alloys Fabricated by Wire-Arc Additive Manufacturing”) investigated the effects of TiC/TiB_2_ nanoparticles on the microstructure and mechanical properties of additively manufactured Al-Mg-Si alloys. Figure 17(c_1_–c_3_) show the optical metallographic (OM) structure of the alloys. It was found that without nanoparticle addition, numerous microcracks were present in the images; however, after nanoparticle addition, the formation of refined, equiaxed grains reduced thermal stresses caused by solidification shrinkage and lowered the likelihood of thermal cracking. Figure 17(d_1_–d_3_) show the EBSD structure of the alloy; the addition of nanoparticles significantly refines the grain structure. Figure 17(e_1_–f_3_) present TEM images of the alloy after T6 heat treatment following nanoparticle addition. The addition of nanoparticles results in the presence of numerous dense, fine, needle-like and spherical Mg_5_Si_6_ precipitates. After T6 heat treatment, the addition of nanoparticles significantly refines the size of the precipitate phases. Figure 17(g_1_–g_4_) summarize the mechanical properties in the as-deposited state and after T6 heat treatment; the addition of nanoparticles significantly improves the alloy’s properties.

Liu et al. [208] reported that biphasic TiC-TiB_2_ nanoparticles accelerated heterogeneous nucleation and inhibited the growth of α-Al, thereby refining the microstructure during solidification. Adding only 0.6 wt.% of biphasic TiC-TiB_2_ nanoparticles to 6061 sheet metal simultaneously improved both strength and ductility. Additionally, Geng et al. [209] found that adding 0.6 wt% and 1.0 wt% TiC reduced the average grain size from 108 μm to 67 μm and 60.1 μm, respectively. Both UTS and YS significantly increased with the addition of nano-TiC particles.

Compared with the findings of Liu and Geng, biphasic nanoparticles yield better refinement effects. This is because, at lower undercooling levels, larger particles in the dual-scale TiC-TiB_2_ system preferentially serve as substrates for α-Al heterogeneous nucleation, generating more grain nucleation than single-phase nanoparticles [210].

**Figure 18:**
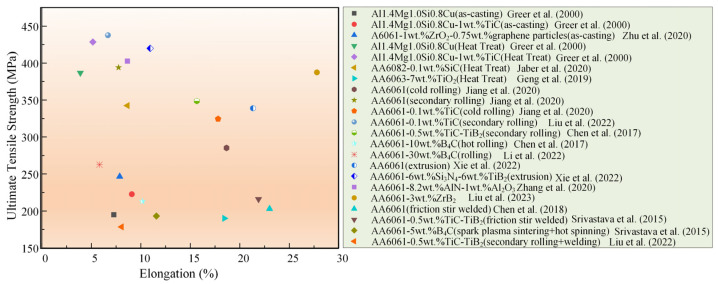
Performance of nanoparticle-enhanced aluminum alloys [208,210,212,214,215,216,217,218,219,220,221,222,223,224,225,226].

TiC nanoparticle addition can also address hot cracking and improve the microstructure of Al-Mg-Si-Cu alloys for enhanced performance [211]. However, effectively introducing TiC into the alloy is a major challenge. Controlling melt temperature is critical for stabilizing TiC in the aluminum matrix, as TiC nanoparticles inevitably react with aluminum below 780 °C to form detrimental by-products such as Al_3_Ti [212]. Al_3_Ti phase degrades mechanical performance, especially ductility [213]. To circumvent this issue, a recommended approach is to synthesize a master alloy via in situ reaction above 800 °C and then add it as nanoparticles to the melt. Zheng et al. [211] demonstrated that adding 1 vol% TiC nanoparticles significantly refined the average grain size, as confirmed by optical microscopy. Additionally, in the as-cast microstructure without nanoparticle addition, severe segregation was observed, whereas after nanoparticle addition, segregation was reduced and the size of the β phase was refined. Specifically, the coarse, interconnected eutectic network became fragmented, thereby improving resistance to thermal tearing. Furthermore, nanoparticle addition enhanced both UTS and EL in both as-cast and processed conditions. Figure 18 shows the properties of nanoparticle-reinforced aluminum alloys.

In summary, nanoparticles can serve as heterogeneous nucleation nuclei for α-Al, thereby refining grain size. Biphasic nanoparticles exhibit better grain refinement effects than single-phase nanoparticles. Additionally, the addition of nanoparticles can improve element segregation within the microstructure and enhance the alloy’s resistance to thermal tearing. Specifically, by introducing nanoparticles into the melt via intermediate alloys, the agglomeration of nanoparticles in the melt can be effectively mitigated, and reactions between nanoparticles and the aluminum matrix can be avoided.

## 6. Strengthening Mechanism of Nanoparticle-Reinforced Aluminum Alloys

**Figure 19:**
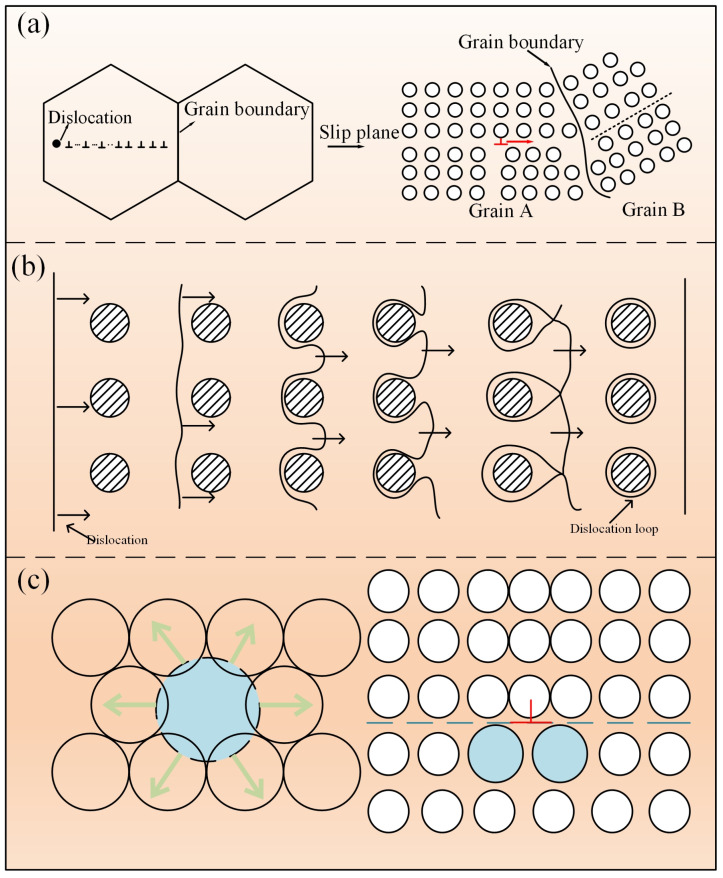
(**a**) Schematic diagram of fine grain strengthening. (**b**) Orowan enhancement schematic. (**c**) Thermal mismatch reinforcement.

The strengthening effect of nanoparticles on aluminum alloys at room temperature is predominantly governed by three mechanisms: Orowan strengthening (Δσ_Orwan_), grain refinement (Hall–Petch) strengthening (Δσ_HP_), and thermal mismatch (CTE) strengthening (Δσ_CTE_). Figure 19 shows a schematic diagram of their reinforcement mechanism. These mechanisms operate individually or synergistically, and their combined contribution can be expressed as

(3) $$\Delta \sigma =\Delta \sigma_{HP}+[(\Delta \sigma_{Orwan}{)}^{2}+(\Delta \sigma_{CET}{)}^{2}{]}^{1/2}$$

(1) Grain refinement strengthening

Grain refinement strengthening improves metal strength by reducing grain size. When nanoparticles are added to an aluminum matrix, they can act as heterogeneous nucleation sites, providing more nucleation sites in the matrix and promoting grain refinement in aluminum alloys during solidification. In addition, nanoparticles can also inhibit grain growth by hindering grain boundary migration. According to the Hall–Petch relationship [226]:

(4) $$\sigma_{y}=\sigma_{0}+\frac{k}{\sqrt{d}}$$

where σ_y_ is the YS; σ_0_ is the YS of the single crystal, representing the resistance to dislocation movement within the grain; k is the Hall–Petch coefficient, representing the hindrance to dislocation movement at grain boundaries; and d is the average grain size. As can be seen from the formula, a decrease in grain size significantly increases the YS of the material. In addition to grain refinement, nanoparticles can refine eutectic phases during solidification and precipitates during heat treatment. During solidification, nanoparticles may serve as heterogeneous nucleation sites for the eutectic phase, representing the most ideal and direct refinement method. However, this requires a coherent or semi-coherent crystallographic relationship between the nanoparticles and the eutectic phase. Consequently, the primary mechanism for eutectic refinement often involves nanoparticle-induced refinement of α-Al grains. These fine α-Al grains segment the residual eutectic melt into numerous isolated micro-pools, restricting the growth space of the eutectic phase and resulting in a fine and dispersed morphology. The strengthening precipitates in aluminum alloys nucleate from a supersaturated solid solution. Heterogeneous nucleation on defects or foreign particle surfaces lowers the nucleation barrier. Artificially added nanoparticles provide abundant heterogeneous nucleation substrates for precipitates. With numerous nucleation sites, each nucleus captures only a limited number of solute atoms, restricting growth space and resulting in extremely fine (often nanoscale) precipitates and avoiding the coarse precipitation that typically occurs at dislocations or grain boundaries.

(2) Orowan reinforcement

Orowan strengthening is achieved by dispersing hard particles in a uniform matrix. When nanoparticles are uniformly distributed in an aluminum alloy, they act as obstacles to dislocation movement. Dislocations must bypass these nanoparticles, thereby increasing the resistance to dislocation movement. According to Orowan strengthening [227], nanoparticles and secondary phases significantly enhance the YS of aluminum alloys by pinning dislocations and impeding their movement, thereby strengthening the aluminum alloy.

(3) Thermal mismatch strengthening

Thermal mismatch strengthening arises from the difference in the coefficient of thermal expansion (CTE) between nanoparticles and the aluminum alloy matrix. During cooling, this mismatch causes internal stresses that generate and multiply dislocations, thereby achieving a strengthening effect [228]. This strengthening mechanism works by increasing the dislocation density. Meanwhile, thermal expansion mismatch strengthening is contingent upon the CTE difference between the nanoparticles and the matrix.

## 7. Summary and Outlook

This paper systematically reviews recent advances in die-cast aluminum alloys, focusing on Al-Si-Mg and Al-Mg-Si series alloys. The review covers alloy design, nanoparticle strengthening, microstructural control, and the underlying mechanisms of property enhancement. It demonstrates through sophisticated alloy composition design, especially the synergistic use of multiple microalloying elements, the effective refinement of grain size along with optimization of the morphology, size, and distribution of strengthening precipitates (e.g., β″, QP, Q′, and η′ phases), thereby achieving an excellent strength–ductility balance in the as-cast state. For example, the introduction of Cu promotes the formation of Al_2_Cu phase or alters the precipitation sequence (e.g., suppressing β″ while favoring QP). The addition of Zn forms MgZn_2_ strengthening phases and increases undercooling, promoting α-Al nucleation and refining the microstructure. Rare earth elements demonstrate outstanding effects on grain refinement, modification of eutectic Si phases, suppression of harmful phases (such as Fe-rich phases), and enhancement of thermal stability due to their unique physicochemical properties. Furthermore, nanoparticle strengthening (such as TiC, TiB_2_, TiCN, SiC, etc.) offers another powerful pathway to elevate the properties of heat-treatment-exempt aluminum alloys. These nanoparticles not only significantly refine α-Al grains and eutectic silicon phases while mitigating macro-segregation, but also introduce numerous dislocation pinning sites through mechanisms like Orowan strengthening, load transfer, and thermal mismatch strengthening, substantially enhancing strength, hardness, and fatigue performance. Notably, the synergistic effects of biphasic or multiphase nanoparticles (such as TiC-TiB_2_) often surpass single-phase particles, enabling more effective control over solidification and microstructural evolution. Advances in preparation techniques, including master alloy methods, stir casting, powder metallurgy, and additive manufacturing, offer diverse solutions to critical challenges such as achieving uniform nanoparticle dispersion, ensuring strong interfacial bonding, and preventing detrimental reactions.

Currently, die-cast aluminum alloys have transitioned from laboratory to industrial applications, successfully implemented in integrated floor panels, front compartments, battery pack housings, and other components. However, the field still faces numerous challenges. First, the complexity of alloy composition design demands a deeper understanding of the interactions between multiple elements and precipitation kinetics to avoid segregation and harmful phases while balancing mutually conflicting performance metrics such as strength, toughness, castability, and hot-tear resistance. Second, industrially scalable addition, uniform dispersion, and precise control of the interface between nanoparticles and the matrix remain technical bottlenecks, while cost is a critical factor for widespread application. Third, for ultra-large thin-walled die-castings, precise control of filling and solidification through mold design, parameter optimization (such as pressure, speed, and vacuum level), and numerical simulation are essential to minimize internal defects (e.g., porosity and shrinkage porosity) and ensure uniformity and consistency in the overall performance of the components. Additionally, for recycled aluminum utilization, enhancing impurity tolerance (especially to iron) and developing high-strength, tough, and environmentally friendly recycled aluminum alloys that eliminate the need for heat treatment are key to achieving a circular economy.

Looking ahead, the development of die-cast aluminum alloys in new energy vehicles will follow several trends. On the one hand, fundamental research will increasingly focus on multi-scale, multi-physics simulations and high-throughput computing, combined with in situ characterization, to reveal the interaction mechanisms among alloying elements, nanoparticles, and the aluminum matrix at the atomic/electronic level. This will elucidate the microstructural evolution during solidification and solid-state phase transformations, providing theoretical guidance for rational design of compositions and processes. Alloy systems will evolve toward diversification and customization, extending beyond Al-Si-Mg and Al-Mg-Si to explore systems such as Al-Zn-Mg and Al-Cu-Mn. Greater attention will be paid to precise Mg/Si ratios, trace element proportions, and the synergistic effects of rare earth elements, aiming for as-cast strength–toughness combinations that match or surpass T6 levels. On the other hand, technological innovation will focus on advancing nano-reinforcement techniques, including novel core–shell structured nanoparticles, surface modification for enhancing interfacial compatibility, and dispersion/structural regulation under external fields (e.g., electromagnetic and ultrasound). Integration of additive manufacturing with nano-reinforcement will enable the integration of component performance and geometry. Concurrently, sustainability will drive greater emphasis on life-cycle assessment, accelerating the development of high-performance recycled high-strength aluminum alloys to reduce reliance on virgin resources and lower carbon emissions. Ultimately, through deep integration and continuous innovation of materials, processes, equipment, and simulation, die-cast aluminum alloys will play an increasingly pivotal role in advancing vehicle lightweighting, extending driving range, and reducing manufacturing costs. They will also provide robust material support for lightweight applications in other high-end equipment manufacturing sectors such as aerospace and rail transit, demonstrating broad application prospects and profound socioeconomic value.

## Figures and Tables

**Table 1:** Die-cast aluminum alloy Al-Si alloys: grades and composition design.

Alloy	Si	Fe	Cu	Mn	Mg	Ti	Sr	Other
Castsil37 [23]	8.5~10.5	0.15	0.05	0.35~0.6	0.06	/	0.01~0.015	Mo, Zr
C611 [24]	4.0~7.0	0.15~0.2	/	0.4~0.8	0.15~0.25	0.1	0.01~0.015	/
Aural 5 [25]	6.5~8.5	0.1~0.25	0.02	0.35~0.7	0.1~0.4	0.15	0.01~0.015	Zn
Tesla Alloy1 [26]	6.5~7.5	0.4	0.4~0.8	0.35~0.7	0.1~0.4	0.15	0.015~0.03	Cr, V
Tesla Alloy2 [26]	6.0~11.0	0.5	0.3~0.8	0.35~0.8	0.15~0.4	0.15	0.015~0.05	Cr, V
Tesla Alloy3 [26]	6.0~11.0	0.5	0.3~0.8	0.35~0.8	0.1~0.4	0.15	0.015~0.05	Cr, V
JDA 1 [27]	8.5~11.5		0.5~3.0	0.1~0.8	0.25~0.5	0.15~0.35		P, RE
Nio Alloy [28]	7.0~9.0	0~0.8		0~0.5		0.1~0.2	0.01~0.03	V, Cr

**Table 2:** Die-cast aluminum alloy Al-Mg alloys: grades and composition design.

Alloy	Si	Fe	Cu	Mn	Mg	Zn	Ti	Other
Magsimal59 [29]	1.8~2.6	0.2	0.03	0.5~0.8	5.0~6.0	0.07	0.2	Be
Castduct42 [29]	0.2		1.5~1.7	0.2	0.15	4.1~4.5	0.2	Be
SJTU-Al-Mg-Cu [26]			0.5~1.5	0.6~0.9	4.5~7.5		0.1~0.2	Be, Re
SJTU-Al-Mg-Si [26]	2~3.6			0.6~0.9	6.0~8.0		0.1~0.2	Be

**Table 3:** Vehicle models and castings applied with heat-free treatment.

Automobile Enterprise	Application Models	Integrated Castings
Tesla	Model Y	Backplane, fore nacelle
HiPhi	HiPhiZ	Rear cabin
Nio	ET5	Backplane
Zeekr	Zeekr009	Rear-end aluminum
Xiaopeng	XiaopengG6	Backplane, fore nacelle
Xiaomi	XiaomiSU7	Backplane

**Table 4:** Physical properties of commonly used ceramic particles [150,151].

Ceramic Particles	Densities (g/cm^3^)	Melting Point (°C)	Knoop’s Hardness (GPa)	Coefficient of Thermal Expansion (10^−6^/°C)	Modulus of Elasticity (GPa)
TiC	6.103	3067	28~36	8.6	660
SiC	3.21	26,107	26	6.63	630
B6C	2.61	2660	27	6.6	6610
Al_2_O_3_	3.107	2066	18~23	8.1	620
TiN	6.60	32,100	16~20	10.3	260
AlN	3.30	2800	12	6.66	363

## Data Availability

No new data were created or analyzed in this study. Data sharing is not applicable.

## Nomenclature

Abbreviation	Definition
HPDC	High-pressure die-casting
ESC	External solidified crystal
CGC	Counter gravity casting
YS	Yield strength
UTS	Ultimate tensile strength
EL	Elongation
TEM	Scanning transmission electron microscope
IGC	Intergranular corrosion
LPBF	Laser powder bed fusion
LDED	Laser-directed energy deposition
TRC	Twin-roll cast
IDC	Integrated die-casting
IDCT	Integrated die-casting technology
